# Lighting the Way to See Inside Two-Photon Absorption Materials: Structure–Property Relationship and Biological Imaging

**DOI:** 10.3390/ma10030223

**Published:** 2017-02-23

**Authors:** Qiong Zhang, Xiaohe Tian, Hongping Zhou, Jieying Wu, Yupeng Tian

**Affiliations:** 1 Key Laboratory of Functional Inorganic Materials Chemistry of Anhui Province, Department of Chemistry, Anhui University, Hefei 230039, China; zhangqiong.314@163.com (Q.Z.); zhpzhp@263.net (H.Z.); 2 School of Life Science, Anhui University, Hefei 230039, China; xiaohe.t@ahu.edu.cn

**Keywords:** organic fluorophores, organic-inorganic nanohybrids, metal complexes, two-photon fluorescence microscopy

## Abstract

The application of two-photon absorption (2PA) materials is a classical research field and has recently attracted increasing interest. It has generated a demand for new dyes with high 2PA cross-sections. In this short review, we briefly cover the structure-2PA property relationships of organic fluorophores, organic-inorganic nanohybrids and metal complexes explored by our group. (1) The two-photon absorption cross-section (*δ*) of organic fluorophores increases with the extent of charge transfer, which is important to optimize the core, donor-acceptor pair, and conjugation-bridge to obtain a large *δ* value. Among the various cores, triphenylamine appears to be an efficient core. Lengthening of the conjugation with styryl groups in the D-π-D quadrupoles and D-π-A dipoles increased δ over a long wavelength range than when vinylene groups were used. Large values of *δ* were observed for extended conjugation length and moderate donor-acceptors in the near-IR wavelengths. The *δ* value of the three-arm octupole is larger than that of the individual arm, if the core has electron accepting groups that allow significant electronic coupling between the arms; (2) Optical functional organic/inorganic hybrid materials usually show high thermal stability and excellent optical activity; therefore the design of functional organic molecules to build functional organic-inorganic hybrids and optimize the 2PA properties are significant. Advances have been made in the design of organic-inorganic nanohybrid materials of different sizes and shapes for 2PA property, which provide useful examples to illustrate the new features of the 2PA response in comparison to the more thoroughly investigated donor-acceptor based organic compounds and inorganic components; (3) Metal complexes are of particular interest for the design of new materials with large 2PA ability. They offer a wide range of metals with different ligands, which can give rise to tunable electronic and 2PA properties. The metal ions, including transition metals and lanthanides, can serve as an important part of the structure to control the intramolecular charge-transfer process that drives the 2PA process. As templates, transition metal ions can assemble simple to more sophisticated ligands in a variety of multipolar arrangements resulting in interesting and tailorable electronic and optical properties, depending on the nature of the metal center and the energetics of the metal-ligand interactions, such as intraligand charge-transfer (ILCT) and metal-ligand charge-transfer (MLCT) processes. Lanthanide complexes are attractive for a number of reasons: (i) their visible emissions are quite long-lived; (ii) their absorption and emission can be tuned with the aid of appropriate photoactive ligands; (iii) the accessible energy-transfer path between the photo-active ligands and the lanthanide ion can facilitate efficient lanthanide-based 2PA properties. Thus, the above materials with excellent 2PA properties should be applied in two-photon applications, especially two-photon fluorescence microscopy (TPFM) and related emission-based applications. Furthermore, the progress of research into the use of those new 2PA materials with moderate 2PA cross section in the near-infrared region, good biocompatibility, and enhanced two-photon excited fluorescence for two-photon bio-imaging is summarized. In addition, several possible future directions in this field are also discussed (146 references).

## 1. Introduction

In 1931, the concept of the two-photon absorption (2PA) process was first proposed by Göppert-Mayer (1906–1972) in her doctoral dissertation [1], which was supervised by Max Born, a Nobel laureate and one of the most distinguished physicists in the twentieth century. In 1961, one year after the invention of the first laser device [2], Kaiser and Garrett reported the first observation of a 2PA-induced frequency-upconversion fluorescence in a GaF_2_:Eu^2+^ crystal sample, excited by the intense coherent radiation of 694.3 nm wavelength from a pulsed ruby-crystal laser [3]. This was the first experimental confirmation of the prediction given by M. Göppert-Mayer 30 years before. Since then, a new major research area of two-photon and multiphoton processes has been opened to scientists and engineers. The main difference between one-photon absorption (1PA) and two-photon absorption (2PA) is that 2PA involves the simultaneous interaction of two photons, and so it increases with the square of the light intensity, whereas 1PA depends linearly on the intensity. This is the reason why 2PA is only observed in intense laser beams, particularly focused pulsed lasers, which generate a very high instantaneous photon density. Most of the applications for 2PA result from this intensity dependence. Two-photon absorption (2PA) is a way of accessing a given excited state by using photons of half the energy (or twice the wavelength) of the corresponding one-photon transition, thus leading to other applications.

There is now a strong demand for efficient 2PA dyes for a wide range of applications, including microscopy [4,5,6,7,8,9,10,11,12,13], micro-fabrication [14,15,16], three-dimensional data-storage [17,18], up-converted lasing [19,20], and photodynamic therapy [21,22,23]. Recently, this demand has been matched by rapid advances in the design and synthesis of 2PA dyes [24,25,26,27,28,29,30,31,32,33,34]. This field has been comprehensively reviewed [35,36,37,38,39,40]. Here we identify the key principles and emerging structure-property relationships, and illustrate these concepts by comparing the behavior of selected chromophores which have been reported by our group. We conclude by discussing the design of two-photon dyes for the most prominent bio-applications. Due to the limited length of this review, only representative examples will be presented, the other related work will be listed in references as thoroughly as possible and readers are encouraged to consult these references for further information.

## 2. Measurement Methods

The 2PA cross-section (*δ*) is the most commonly used parameter for characterizing 2PA fluorophores; *δ* values can be strongly influenced by the measurement techniques. The two main techniques for measuring 2PA cross-sections are known as Z-scan technique and two-photon excited fluorescence (2PEF). The other techniques which provide less direct information on 2PA cross-sections (such as degenerate four-wave mixing) or which are less widely used (such as the white-light continuum method, fs-WLC) are beyond the scope of this review.

### 2.1. The Z-Scan Technique

**Figure 1:**
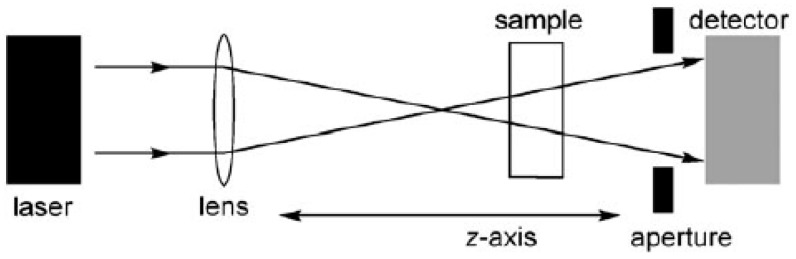
Experimental setup for the Z-scan experiment.

The Z-scan technique involves moving a sample along the path of a focused laser beam and measuring the light intensity at the detector as a function of its position along this *z*-axis, as summarized in Figure 1 [41]. If the detector has a narrow aperture (as in the so called “closed-aperture” setup), then the output is sensitive to intensity-dependent changes in the refractive index (as a result of third-order nonlinear polarizability or thermal effects) which lead to self-focusing or defocusing of the beam. Alternatively, if the detector collects all the light from the sample (“open-aperture” setup), then the output only reflects the intensity-dependent transmission, and can be used to measure 2PA cross-sections. Two effects other than true simultaneous two-photon absorption can contribute to the apparent 2PA cross-sections measured by open-aperture Z-scan experiments [42].

(1) Light can be lost due to self-defocusing (if the aperture of the detector is too narrow or too far from the sample) or because of nonlinear scattering; this results in extra contributions to the apparent nonlinear absorption.
(2) A built up of excited-state populations (by either one-photon or two-photon absorption) can lead to nonlinear transmission through excited-state absorption (ESA). The contribution from ESA can be reduced by the use of wavelengths where there is negligible 1PA, very short laser pulses (<1 ps), and low repetition rates; a repetition rate of less than 1 kHz may be needed to allow excited triplet states to fully decay between pulses.

The Z-scan technique is very useful for probing nonlinear transmission and for characterizing nonlinear refraction (using a closed aperture). However, the two problems mentioned above cannot be avoided which could lead to enhance the apparent 2PA cross-section. For example, it has been shown that thermal lens effects can lead to substantial artifacts in 2PA cross-sections measured by the Z-scan technique, even when using femtosecond pulses, a 1 kHz repetition rate, and a wavelength at which there is negligible one-photon absorption.

### 2.2. Two-Photon Excited Fluorescence Method (2PEF)

The 2PEF provides direct information on the efficiency of 2PA. Several variants of this experiment have been developed since it was first reported by Xu and Webb [43]. And the 2PEF technique has been optimized extensively by Rebane, Drobizhev, and co-workers [44]. Recently, they reported accurate reference 2PA spectra for a wide range of commercially available dyes, thus making the 2PEF method particularly attractive [45].

The 2PA cross-section *δ* values of samples were determined by the following Equation:

$$\delta_{s}=\frac{\delta_{r}\times F_{s}\times \varphi_{r}\times C_{r}\times n_{r}}{F_{r}\times \varphi_{s}\times C_{s}\times n_{s}}$$

Here, the subscripts ref. stands for the reference molecule, *δ* is the 2PA cross-section value, *c* is the concentration of solution, *n* is the refractive index of the solution, *F* is the 2PEF integral intensities of the solution emitted at the exciting wavelength, and *φ* is the fluorescence quantum yield. The *δ*_ref_ value of the reference was taken from the literature [46,47].

**Figure 2:**
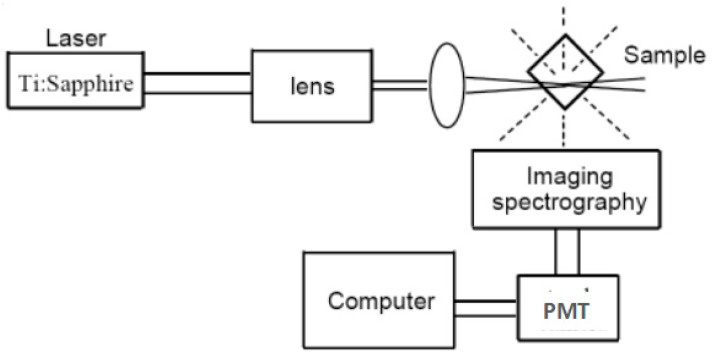
Experimental setup for two-photon fluorescence intensity at different excitation wavelengths.

The 2PEF experiments (Figure 2) require the use of a pulsed laser, typically about 100 fs, although in contrast to the Z-scan method, the accuracy of *δ* values from 2PEF is not strongly dependent on the pulse width [48]. As with most fluorescence measurements, a dilute solution is used (with an optical density of about 0.1), so small amounts of material are required. The intensity of the 2PEF signal increases with the square of the laser intensity; it is important to check this quadratic power dependence, to avoid overestimating the *δ* value because of fluorescence contributions from 1PA. Thus, two limitations of this technique are: (1) it cannot be applied in spectral regions with one-photon absorption and (2) the sample must be photo-luminescent. However, the first of these restrictions is general to all techniques for measuring reliable two-photon cross-sections. The second restriction can be overcome in some cases by quantifying a secondary photochemical process, such as the luminescence generated by energy transfer from the 2PA-generated excited state of the chromophore. In practice, 2PA cross-sections from Z-scan measurements (even using femtosecond pulses) often appear to be exaggerated when compared with 2PEF values [49,50]. Here we focus, where possible, on values from femtosecond two-photon fluorescence (fs-2PEF). The errors in the determination of the 2PA cross-sections are generally greater than 10%, even under the best experimental conditions.

## 3. Design Strategies, Structure-Property Relationships, and Biological Applications

### 3.1. Organic 2PA Fluorophores

Extensive research has been conducted to develop organic materials with large 2PA cross sections for possible applications in optical limiting, nano-fabrication, photodynamic therapy, and optical imaging [51,52]. The results of structure-property relationship studies have established that the 2PA cross sections (*δ*) of donor-acceptor (D-A) dipoles and D-π-D and D-A-D quadrupoles can be increased by using strong D-A groups while maintaining the planar structure to facilitate intramolecular charge transfer (ICT). Based on the discussion of the molecular design for small organic 2PA systems in the preceding section, organic molecules that show two-photon activity can be grouped into three general classes: (i) dipolar, A-π-D; (ii) quadrupolar, A-π-A; D-π-D; A-π-D-π-A; D-π-A-π-D; and (iii) octupolar; 3-branched, A_3_-(D-core) and D_3_-(A-core). Many research groups have synthesized and characterized 2PA-active materials more or less along these guidelines of molecular design, and there is also an inherent difficulty in comparing 2PA cross section results obtained from different measurement techniques and under different experimental conditions.

#### 3.1.1. Pyridinium Derivatives

**Figure 3:**
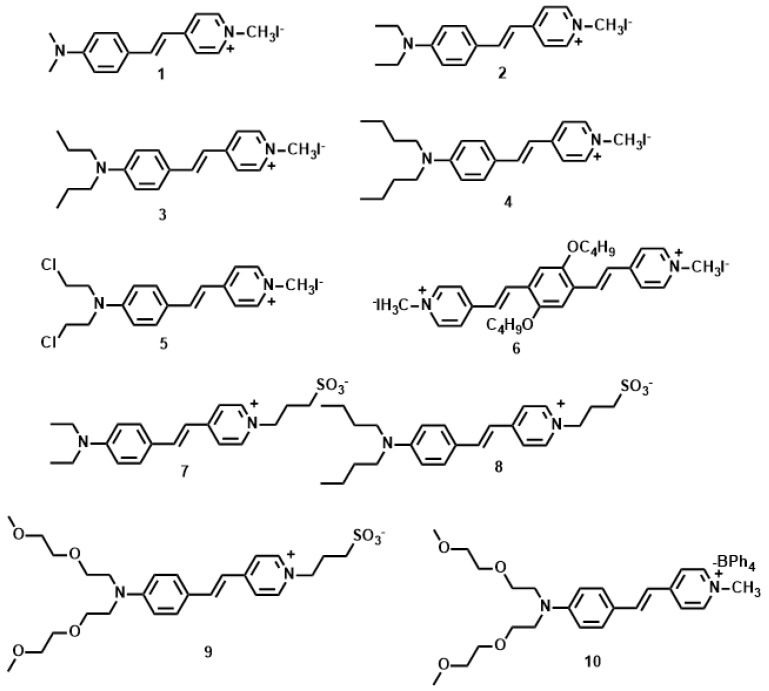
Molecular structures of **1**–**10**.

The *δ* values of the pyridinium derivatives (**1**–**10**) [53,54,55,56,57] increased as the conjugation length was increased by styryl groups, reaching the maximum *δ* value of 309 GM for **6** and the minimum *δ* value of 14.8 for **5** (Figure 3, Table 1). When the conjugation length was increased by a styryl group (**6** vs. **1**–**5**, the *δ* value increased more than three-fold. The molecular structures indicated that **1**–**5** are D-π-A dipoles, whereas as **6** is a A-π-D-π-A quadrupole. *δ* changes little due to the similar donor ability when the donor was changed from Me to *n*-Bu group (**1**–**4**, **7** and **8**). Whereas the OC_4_H_9_ group in the core increase *δ* value significantly, presumably due to the auxiliary electron donating property leading to enhanced ICT. On the other hand, Cl atom at the terminal position signicantly decreased *δ* values (**5** vs. **1**–**4** and **6**), indicating the importance of donating ability in two-photon materials. From **7** to **9**, the growth of the alkyl chain increases the electron density of the bridge, favoring the ICT along the extended *π*-bridge from terminal donor to acceptor group. In the case of **9**, the presence of two methoxyethoxyethyl groups enhances the steric bulk which can hinder π-π stack. Therefore we inferred that not only the electron donating ability of the terminal group impacts the 2PA behavior but the steric bulk as well, that is to say, the steric bulk of the electron-donating terminal group plays a major role in 2PA behavior. As observed for sulfonate salts, the *δ* value increased with the accepting ability compared to methyl pyridinium. (**2**, **4** vs. **7**, **8**). The *δ* value of **10** was four times larger than that of sulfonate salts when the accepting ability of the terminal group was further increased by the stilbazolium group. This result shows the importance of strong ICT over a suitable donor, acceptor, and conjugated length to achieve a large two-photon cross section. Moreover, **9** shows *δ*_max_ values above 100 GM by using the optical excitation wavelength at 960 nm, which are the most attractive features for its utility as a biological probe.

#### 3.1.2. Pyrimidine Derivatives

Pyrimidine derivatives were intensively investigated as electron luminescent materials in the past [58,59] owing to the *π*-electron deficient and planarity they provide. The compound 4,6-dimethylpyrimidine was selected as the central unit, which allows tuning of the electronic delocalization along the conjugated backbone in the ground state by modulation of the twist angle between the two halves of the molecules [60].

**Figure 4:**
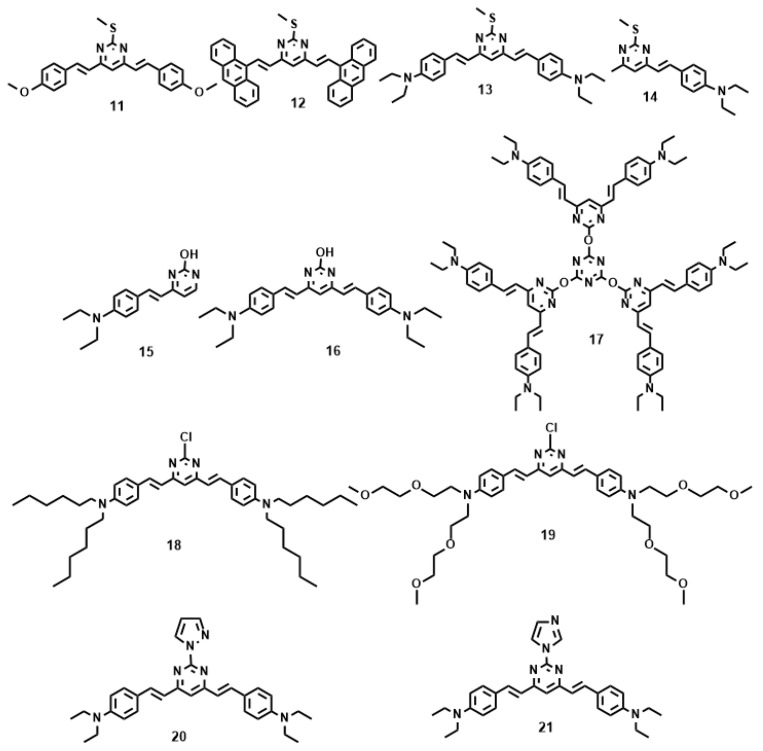
The molecular structures of **11**–**21**.

**Figure 5:**
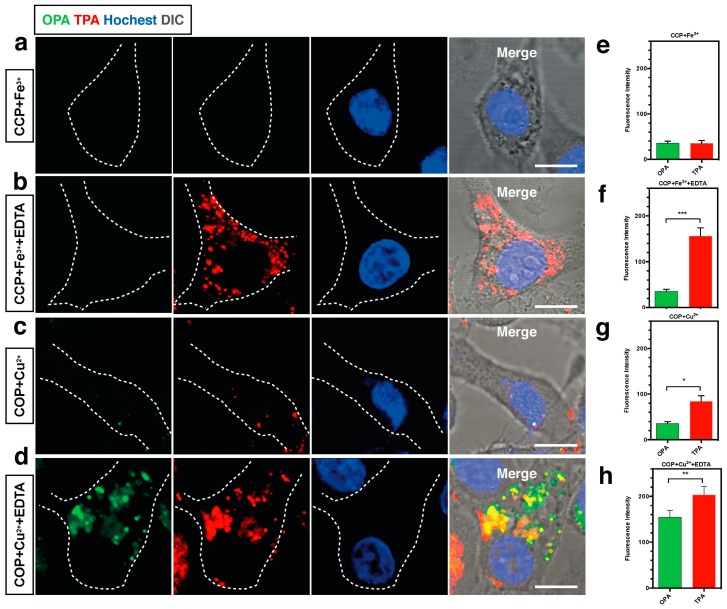
(**a**) One and two-photon florescence image of HepG2 cells with **18** in the presence of Fe^3+^ (*λ*_ex_ = 840 nm, emission wavelength from 575 to 675 nm); (**b**) One and two-photon florescence image of HepG2 cells with **18** in the presence of Fe^3+^ and EDTA (*λ*_ex_ = 840 nm, emission wavelength from 575 to 675 nm); (**c**) One and two-photon florescence image of HepG2 cells with **19** in the presence of Cu^2+^ (*λ*_ex_ = 820 nm, emission wavelength from 575 to 675 nm); (**d**) One and two-photon florescence image of HepG2 cells with **19** in the presence of Cu^2+^ and EDTA (*λ*_ex_ = 820 nm, emission wavelength from 575 to 675 nm); (**e**–**h**) Normalized fluorescence intensity analysis corresponding to (a–d), cell number *n* = 30, one-way ANOVA was used for statistical analysis for independent experiments, *p* < 0.005.

Figure 4 and Table 2 show the values of *δ* of chromophores **11**–**21** in dimethylformamide (DMF). As noted from Table 2, increasing the strength of electron-donating end-groups results in a pronounced enhancement of the 2PA cross sections in the NIR region. The highest values of *δ* are 8 for **11**, 10 for **12**, 498 for **13,** and 104 for **14** [61]. A similar effect is observed by increasing the branch number, the 2PA cross-sections show the sequence: **17** > **16** > **15** [62]. Compared with **15**, **16** bearing the much larger 2PA cross-section may imply that extending the *π*-conjugation domain, accomplished by increasing the number of branches of a chromophore molecule based on the structural motif of a multi-branched olefin, could be a useful approach toward enhanced nonlinear absorptivities [63,64]. With respect to **17**, an oxygen atom as the linkage was adopted to combine the 1,3,5-triazine core with substituents to accomplish a large 2PA cross-section (1885 GM), however the 2PA cross-section of **17** is much lower than three times the cross-section of **16**. There is no obvious enhancement when going from **16** to **17**, not even an additive effect. It is to be noted that the pyrimidine derivatives of **18** and **19** having hexyl and methoxyethoxyethyl as the end donor groups have been studied as two-photon sensors for metal ions in CH_3_CN-H_2_O [65]. The **18** + Fe^3+^ system shows a 20-fold 2PA enhancement in response to EDTA, as well as the **19** + Cu^2+^ system which shows an approximately 7-fold 2PA enhancement in the presence of EDTA. Further cell TPM micrographs showed these two novel probes could selectively detect Fe^3+^ and Cu^2+^ in live cells in a very sensitive way without interference from the other metal ions, shown in Figure 5. Comparison of the 2PA properties reveals the relative efficiency of the cores. The values of *δ* of **21** are smaller than **20** [66], understandably due to the acceptors linked to the cores (Figure 4). *δ* increased by six times, when the core was changed from pyrazolyl (**20**) to imidazolyl (**21**). This indicates that the relative efficiency of the core in the D-π-A-π-D quadrupoles decreases in the order, –OH > pyrazolyl > imidazolyl, which are prone to the ICT progress in the D-π-A-π-D system, leading to stronger 2PA.

#### 3.1.3. Triphenylamine Derivatives

**Figure 6:**
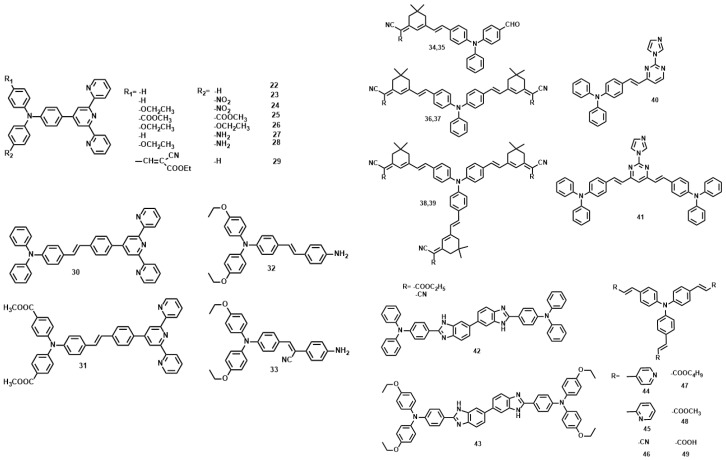
The molecule structures of **22**–**49**.

P. N. Prasad, firstly reported that multi-branched structure significantly increases the 2PA cross section in comparison to the single-branched counterpart [67]. In the past decade, triphenylamine has been extensively employed as the core due to its capability of extending the conjugation length through the propeller structure [68,69,70,71,72]. Comparison of the 2PA properties reveals the relative efficiency of the cores. The *δ* values for **25** [73] and **26** [74] are smaller than **22**, **27**, and **28**, understandably, due to the twisted D-A structures and different donor groups (Figure 6, Table 3). When the core was changed from triphenylamine to the triphenylamine vinylene group, *λ*_max_ (2PEF) was red-shifted by more than 20 nm and *δ* increased a lot. This indicates that the relative efficiency of the core in the D-π-A dipoles increases in the order, **30** > **31**. On the other hand, CN groups at the vinylic positions significantly influence *δ* values (**29** [75] vs. **22**–**28**), indicating the importance of planarity in two-photon absorption materials. The change of the end groups from terpyridine to aniline decreased the *δ* value more than three fold (**32**, **33** [76] vs. **30**, **31**) with a concomitant blue shift in *λ*_max_ (2PEF). This indicates that ICT is more efficient in D-π-A dipoles than in D-π-D quadrupoles, hence a stronger 2PA. The value of *δ* for **34**–**39** [77] increased from 660 to 9398 GM as the amount of branching increased. A theoretical study revealed that such an enhancement is mainly caused by the vibronic coupling [78]. The electronic coupling is weak, probably because the central amino group is used as the connecting unit, which breaks the conjugation of the whole network. On the other hand, increasing the length of the conjugation bridge will increase the density of state, providing more effective coupling channels, which would in turn increase the 2PA cross section. A similar result was observed in the pyrimidine-triphenylamine derivatives (**40**, **41** [79]). Incorporation of hetero-cyclic rings into the triphenylamine system caused a significant blue-shift in the absorption, emission and two-photon absorption maxima (*λ*_max_ (2PEF)) (**42**, **43** [80] vs. **44**–**49** [81]), possibly due to the reduced planarity around the double bond. However, except for the lower quantum yield in the heterocyclic analogs, no systematic change was observed in the *δ* values. As observed in most D-A-D quadrupoles, the *δ* value increased by changing the stronger donor strength, ethyloxy triphenylamine as the terminal group (**42** vs. **43**).

### 3.2. Organic-Inorganic Nanohybrids

Over the past few years, increasing attention has been devoted to understanding the interactions between organic and metallic components in organic-metal nanohybrids [82,83,84,85], which can greatly improve the overall application performance of the nanohybrids, combine different properties into one material (such as electronic, magnetic, optical properties, and so on), and bring about new physical and/or biological properties [86,87]. Moreover, it has long been known that the structures at the organic-metal interface have some important influence on the properties of the nanohybrid. As a consequence, the interaction mechanisms between the different components in nanohybrids are important for understanding the functions of these materials and for the rational design of a functional nanohybrid. Cohanoschi et al. [88] observed strong surface plasmon enhancement of 2PA of chromophores in solution containing gold colloid. Marder and co-workers [89,90] also observed strong enhancement of the 2PA of organic molecules near silver nanoparticle fractal clusters, and this enhancement effect was manifested in the composite materials with very strong 2PA. Prasad and co-workers [91,92,93,94] reported a novel class of dye-concentrated composite nanoparticles with enhanced 2PA by nano-aggregation. Our previous work [95] found enhanced 2PA by combination of the large organic salt with CdS nanoclusters. This field has been growing rapidly during the past ten years due to the realization of the two-photon technologies as mentioned above and improved characterization methods. All these interesting results are of scientific significance and technical potential.

**Figure 7:**
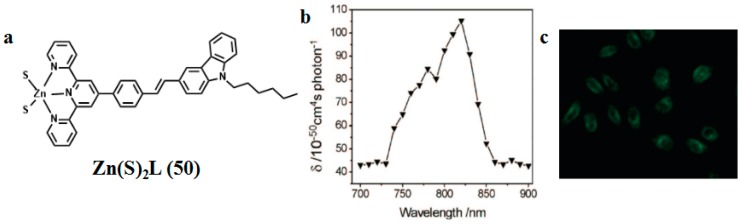
(**a**) The structure of **50** (**b**) Two-photon (200 fs, 76 MHz Ti: sapphire laser) absorption cross section (*δ*) of **50** in DMF versus excitation wavelengths with 380 mw power (**c**) 2PEF image of HeLa cells with excitation at 800 nm.

In 2009, we utilized a facile solvothermal reaction process toward the target Zn(II) complex. ZnS nanocrystals were used as both a zinc source and sulfur source [96]. The selected bulky organic ligand was *N*-hexyl-3-{2-(4-(2,2′:6′,2″-terpyridin-4′-yl)phenyl)ethenyl}-carbazole (labeled as L in Figure 7). The bulky L is a highly *π*-conjugated chromophore, which contains a terpyridinyl tridentate chelate group [97], a two-photon active carbazole moiety [98], a flexible hexyl chain, and a phenylethenyl group as a *π*-conjugated bridge. Significantly, the two sulfur atoms in the complex are identical in the minus one oxidation state (S^1−^) and coordinated to Zn(II) as terminal ligands, which was unknown previously in Zn(II) complexes. The unique feature in this new reaction design is the use of ZnS nanocrystals as a precursor and chromophoric L as an ancillary ligand. In the measured range (700–900 nm), the maximal 2PA cross section (*δ*) is 105.2 GM at 820 nm, which is suitable in the biological field. Two-photon fluorescence microscopy (2PFM) confirmed that the molecules of Zn(S)_2_L (**50**) are presented within the cells, the cytoplasmic distribution is more uniform, and the distribution in the nucleolus is significantly lower. This result demonstrates that the observed cytoplasm uptake must be due to the presence of the intact Zn(S)_2_L (**50**) complex. Thus, the novel structural mode of the Zn(S)_2_L complex and its 2PEF imaging should be intrinsically interesting to chemists, biologists, and material scientists. Particularly, the versatility of the two unsaturated coordinated sulfur atoms could be expected to endow Zn(S)_2_L (**50**) significant promise not only for biomedical applications but also as a “complex” ligand for the surface modification of nanomaterials, even for the synthesis of novel hetero metal complexes.

**Figure 8:**
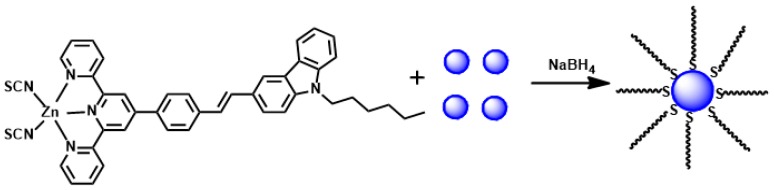
The synthesis route of S-Au NPs (**51**).

Based on the above work, a novel nanostructure material (**51**, Figure 8) [99] was designed by the terminal sulfur of the S ((*E*)-3-(4-([2,2′:6′,2″-terpyridin]-4′-yl)styryl)-9-hexyl-9H-carbazole Zn(SCN)_2_ complex) modified with Nano gold. It could be used to target mitochondria under two-photon laser confocal microscopy, due to the energy of laser irradiation gained from the absorbed photons to be dispersed as excess heat to the neighboring particles and thus to induce their fusion, compared with free complex. Fortunately, **51** has been obtained with longer lifetime, two-photon absorption (2PA) coefficient (*β*), 2PA cross section (*δ*), nonlinear refractive index (*γ*) and third-order nonlinear optical susceptibility (*χ*^(3)^) than the original complex in the near-infrared region (NIR) (Table 4), due to the laser irradiation induced charge transfer from the complex to the gold nanoparticles. Compound **51** can be successfully applied as a two-photon fluorescent probe for labeling the cellular mitochondria in HepG2 cells.

**Figure 9:**
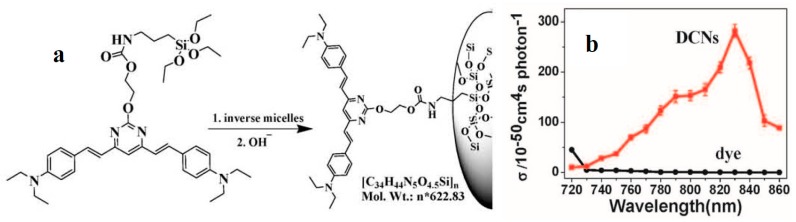
(**a**) Schematic representation of the preparation progress of dye-concentrated nanoparticles (DCNs) (**52**); (**b**) 2PA cross section of dye and DCNs in DMF versus excitation wavelengths from 720 nm to 860 nm in DMF.

Of late, the functional precursor of the dye-concentrated nanoparticles (DCNs) (**52**) [100] was synthesized by covalent linking between **16** and a silane unit 3-isocyanatopropyltriethoxysilane (ICTES) (Figure 9a). We have demonstrated that **52** results in a solid holding of the linear optical properties of the dye, which demonstrates the utility of using the silica particles as a tool for concentrated dyes. The *δ* of the free dye is negligible in the absence of DCNs but is strongly enhanced in DCNs (*δ* = 284 GM) (Figure 9b). It should be mentioned that dye **16** does exhibit a cross-section of 1319 GM, whereas nanoparticles exhibit a cross-section of 284 GM. Therefore, there is really a 4-fold decrease when incorporating dyes **16** within nanoparticles, even if the cross-section of the intermediates is negligible. It is worth noting that the *δ* value of **52** exceeds that of many fluorophores widely used in biology, including fluorescein, BODIPY, DAPI, and GFP [101]. The functional DCNs (**52**) act as a guide to the design of more efficient 2PA materials correlating well with the presence of organic chains in the host inorganic framework as well as applicable to a broad range of modified fluorophores widely used in biology.

Silver NPs have been widely studied in a number of applications such as catalysis [102], photocatalysis [103], sensing [104], surface enhanced Raman scattering (SERS) [105,106] and biomedical fields [107,108,109,110]. Silver in low concentration is nontoxic to human cells. Since ancient times, silver has been extensively employed to control spoilage and light infections. In recent years, the antibacterial and antiviral actions of Ag NPs have been thoroughly investigated [111]. Moreover, the physical, chemical, and biological properties of Ag NPs can be designed and tuned by changing the size, shape, and surface agents. Thiols, carboxylates, amides, imidazoles, indoles, hydroxyls, and DNA oligo-nucleotides [112,113] are common organic compounds utilized to prepare Ag-based nanostructures, among which the interactions between Ag and S atoms are very strong. As mentioned in the last section, we have devoted considerable effort to select organic building blocks for enhanced two photon absorption properties. However, little literature has reported the dye/Ag nanohybrid used as two-photon (2P) probes for specific applications, such as biological imaging by using 2PFM [114,115]. Thus, there is a vast space to develop efficient dye/Ag nanohybrid 2P probes with large 2PA for in vivo 2PFM imaging.

**Figure 10:**
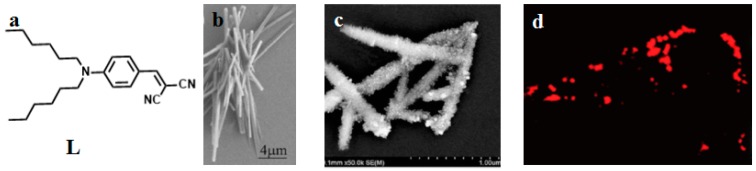
(**a**) Schematic representation of L; (**b**) SEM (Scanning electron microscope) micrograph of L nanorods prepared from EG (ethylene Glycol) solution; (**c**) SEM micrograph of the nanohybrid (**53**); (**d**) Two-photon fluorescence Image of HepG2 cells incubated with 20 μm of **53** for 30 min, then washed with PBS. *λ*_ex_ = 760 nm (emission wavelength from 520 to 562 nm).

Considering all the above aspects, 2-(4-(dihexylamino)-benzylidene)-malononitrile (L) (Figure 10) [116], was used to couple with AgNCs, as the hexyl chain could be employed as both electron-donor unit and a flexible chain to increase the solubility of the compound. The dicyano group was used as an electron-acceptor unit, and the two units were linked by a vinyl bond to form a D-π-A structure. L coupled with Ag through electron-acceptor units to form the nanohybrid (**53**), which consisted of AgNCs approximately 20 nm in diameter uniformly dispersed on the surface of nanorods of L. The hybrid then self-assembled to form nanorods. The coupling effect also resulted in enhancement of a series of nonlinear optical properties, including 2PEF, 2PA cross section (*δ*), 2PA coefficient (*β*), nonlinear refractive index (*γ*), and third order nonlinear optical susceptibility (*χ*^(3)^) (Table 5). The enhanced two photon fluorescence of the nanohybrid (**53**) was proven to be useful for 2PFM imaging in live cells, such as HepG2. The results show that the nanohybrid is cell-permeable and suitable for cytoplasm staining and uptake.

### 3.3. Metal Complexes

#### 3.3.1. Lanthanide-Diketonates

**Figure 11:**
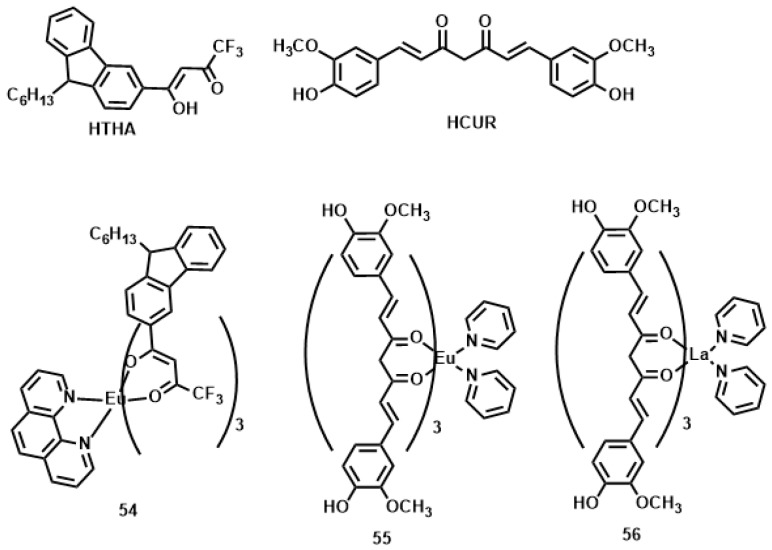
The structures of ligands HTHA (4,4,4-trifluoro-1-(9-hexylcarbazole-3-yl)-1,3-butanedione) and HCUR (1,7-bis(4-hydroxy-3-methoxyphenyl)-1,6-heptadiene-3,5-dione) and its rare earth complexes **54**–**56**.

**Figure 12:**
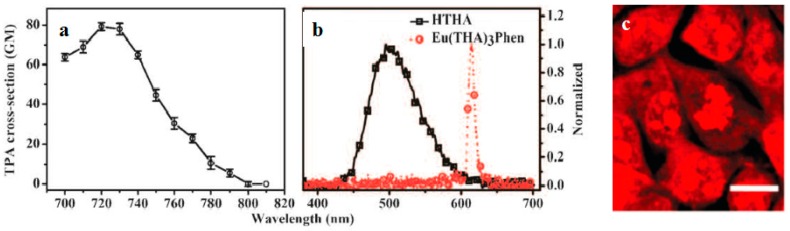
(**a**) 2PA cross sections (*δ*) for Eu(THA)_3_Phen (**54**); (**b**) Two-photon-excited fluorescence (2PEF) of HTHA and Eu(THA)_3_Phen (**54**) in DCM (1 × 10^−3^ mol·L^−1^); (**c**) Live cellular image based on Eu(THA)_3_Phen (**54**), MCF-7 cells were incubated with 200 μM complex for 1 hour, then imaged by two-photon microscopy (excitation wavelength *λ* = 770 nm, emission wavelength *λ* = 613 nm) without fixation. Note that cell cytosol staining is clearly emerging in higher concentration; in contrast cell nucleus and nucleoli luminescence is more significant in low concentration. All the scale bars represent 10 mm.

**Figure 13:**
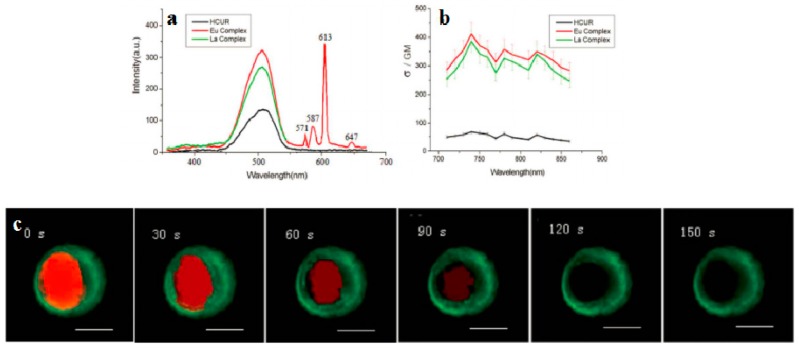
(**a**) Two-photon-excited fluorescence (2PEF) of HCUR, **55** and **56** in DMF (1 × 10^−3^ mol·L^−1^); (**b**) 2PA cross sections (*δ*) for HCUR, **55** and **56**; (**c**) The fluorescent imaging of the MCF-7 cells labeled with the **55** (green) and PI (red) at different times under continuous light exposure (all the scale bars represent 10 μm).

Lanthanide-diketonates are complexes of *β*-diketone ligands (1,3-diketones) with lanthanide ions. These complexes are the most popular and the most intensively investigated luminescent lanthanide coordination compounds. Their popularity is partially because many *β*-diketones are commercially available and the synthesis of the corresponding lanthanide complexes is relatively easy as well because of their excellent luminescence properties. In several reviews on luminescent lanthanide compounds, the *β*-diketonate complexes are described [117,118]. Because of their frequent use in lanthanide-based luminescent hybrid materials, some of the general properties of the lanthanide *β*-diketonate complexes will be discussed in this section. A difference is made between the *β*-diketone and the corresponding *β*-diketonate ligand which is obtained by deprotonation of the *β*-diketone. The europium (Eu(III)) and lanthanum (La(III)) *β*-diketonate complexes often show an intense luminescence, the luminescence intensities are strongly dependent on the type of *β*-diketone and on the type of complexes. However, ultra-violet light is usually used for the sensitization of lanthanide complexes, which limits the investigation depth and presents some phototoxicity due to inherent features of this high energy excitation. Acknowledgedly, the 2PEF technique is desirable to overcome this obstacle whose light located in the near-IR range provides higher resolutions, lower photodamage, and photobleaching in imaging. Therefore, developing the two-photon sensitizable lanthanide complexes could combine precisely the above advantages into one probe. To achieve effective two-photon sensitized luminescent probes, the “antennae” with efficient 2PA for light-harvesting are needed to overcome the poor extinction coefficients of the lanthanide ions caused by the symmetry-forbidden nature of the inner-shell f-f Transition [119,120]. Initially, the “antenna” effects were utilized to sensitize the luminescence of Eu(III) and La(III), which were to explore directly the cellular uptake and localization characteristics in live cells. A list of the most often used *β*-diketones in our group is given in Figure 11. These intrinsic biological fluorophores chelated to Eu(III) and La(III) generally give moderate 2PA activities, whereas they provide a new challenge to make new two-photon-sensitizable lanthanide probes for bio-imaging. The excitation power dependence was examined for 700–810 nm and used in the subsequent determination of the 2PA cross-sections (*δ*), which shows that the Eu(THA)_3_Phen (**54**) [121] is considered to have high efficiency in two-photon sensitization (Figure 12). The maximum *δ* value is estimated to be 80 GM at 720 nm, and notably, the experimental points of 2PA spectra agree with the wavelength-doubled linear absorption spectra of HTHA, which indicates that the sensitized luminescence at 614 nm is attributed to europium [122]. In fact, the THA anion is a two-photon absorber that partakes in energy transfer to the lanthanide, which is the emitter. Additionally, it functions as a luminescent cellular DNA stain being successfully taken up by live MCF-7 cells and clearly displaying nucleus structure. As presented in this work, **54** combines the advantages of two-photon sensitization and lanthanide luminescence well. As for complexes Eu(III) complex (**55**) and La(III) complex (**56**) [123], they possess good luminescence characteristics (high color purity and high sensitivity). The experimental results showed that the optimal excitation wavelengths of **55** and **56** all locate at 740 nm in DMF (Figure 13). The highest cross-section values of *δ* are 412 and 372 GM, respectively. The wavelengths are suitable for bio-imaging in the near IR range. To further clearly verify their fluorescence stability as fluorescent cellular probes, MCF-7 cells were labeled with both **55** and a nuclear dye propidiumiodide (PI, a commercially available organic dye). It shows the bright green colored **55** outside the nuclei and the red-colored dye PI inside the nuclei, respectively (Figure 13), which were simultaneously monitored by the fluorescence of the complex and PI in the same cells with the same continuous light exposure (480 nm). The green fluorescent signal of **55** is very stable against photobleaching throughout the imaging period of 150 s compared with PI (the red fluorescence signals of PI disappeared in 90 s). The results also show that the complex is very stable. As presented in that work, the lanthanide-diketonate complexes combine the advantages of two-photon sensitization and lanthanide luminescence well. Once more this identifies the promising direction for the synthesis of two-photon sensitized luminescent probes for less harmful and better quality bio-imaging.

#### 3.3.2. Transition Metal Complexes

##### Precious Metal Complexes

Precious metal complexes, including Ru, Rh, Pd, Os, Ir, Pt, Ag, Au complexes, have been shown to be the most important materials with phosphorescent emission at room-temperature [124,125,126]. The possible excited states of precious-metal complexes include metal-to-ligand charge-transfer (MLCT), intraligand charge-transfer (ILCT), ligand-to-ligand charge-transfer (LLCT), metal-centered (MC) excited states, metal-metal-to-ligand charge-transfer (MMLCT), ligand-to-metal-metal charge-transfer (LMMCT) and metal-to-ligand-ligand charge-transfer (MLLCT) states. The application of phosphorescent precious-metal complexes as bio-imaging probes is a new research field, but is exhibiting rapid development [127,128,129,130]. Furthermore, some new applications of phosphorescent precious-metal complexes with 2PA property in targeted two-photon fluorescence bio-imaging and small-animal bio-imaging in vitro and in vivo also appeared [131]. In this section, we summarize recent results concerning the use of phosphorescent precious-metal complexes with Ru(II) and Pt(II) for two-photon bio-imaging. In particular, we consider a rational approach to their design and discuss how to obtain stained cellular compartmentalization profiles and how these complexes interact with the intracellular functional molecules.

Despite holding pride of place in the field of strong luminescence, Ru(II) polypyridyl (N^∧^N^∧^N or N^∧^N^∧^C) complexes, are not very well-suited to live cell 2PFM owing to poor cell uptake. Although imaging can be achieved by using transfection agents, detergents, or electroporation to facilitate membrane permeation, these methods are undesirable for live cell samples [132]. On the other hand, dead or non-viable cells have compromised cell membranes and, indeed, it was shown over a decade ago that the famous “DNA light-switch” complex [Ru(bpy)_2_(dppz)]^2+^] [133], whose emission is strongly enhanced upon binding to DNA, could be used as a dead cell marker in flow cytometry and confocal microscopy [134]. The action is similar to that of more conventional dead-cell markers, such as propidium iodide, relying on the ingress into dead cells through compromised membranes, and subsequent binding to DNA with enhancement of emission.

**Figure 14:**
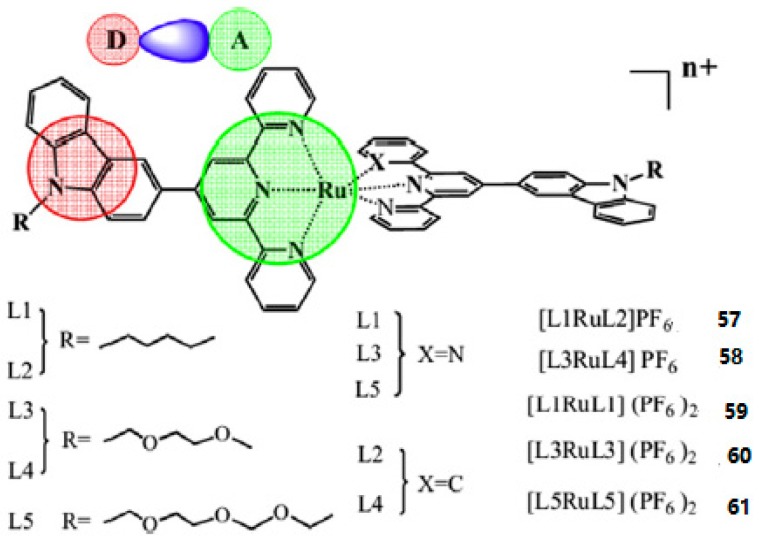
Molecular structures of Ru(II) polypyridyl complexes **57**–**61**.

Based on the above information, Xiao et al. reported five novel Ru (II) polypyridyl complexes (**57**–**61**) (Figure 14) [135] based on donor-acceptor (D-A) ligands, in which the 2,2′:6′,2″-terpyridine or 6-phenyl-2,2′-bipyridine acts as acceptor units (A) and a carbazole moiety as a donor group (D). The alkyl or alkoxy as the auxiliary group connecting to the N atom of the carbazole is to improve the solubility of the metal complexes. The 2PA properties of the complexes were measured by Z-scan techniques and the interaction with DNA by use of optical spectroscopic and viscosity measurements. Table 6 shows the third-order nonlinear parameters of the five complexes, the *δ* values of the complexes **1**–**5** exhibits the order of **59** < **60** < **61** < **57** < **58**.

The *δ* of **57** and **58** are obviously larger than non-cyclometalated Ru(II) complexes **59**–**61** due to the C-Ru bond formation. Because of the steric bulk which can prohibit the interactions of the complex molecules, *δ* is larger than those of complexes which have the smaller terminal group under a similar condition. Then the interactions between complexes and ct-DNA are intercalation mode with varying binding affinities by UV-vis absorption titrations, EB competitive assays, CD spectra, and viscosity measurements discussed in detail. The complexes cannot change the ct-DNA conformation, base stacking, and helicity when they interact with ct-DNA. Complexes **57**–**59** are classical intercalation model but complexes **60** and **61** are partial or nonclassical intercalation model. The results suggest that the five Ru(II) polypyridyl complexes have potential applications in 2PA materials and biological sciences ingress into dead-cell markers.

**Figure 15:**
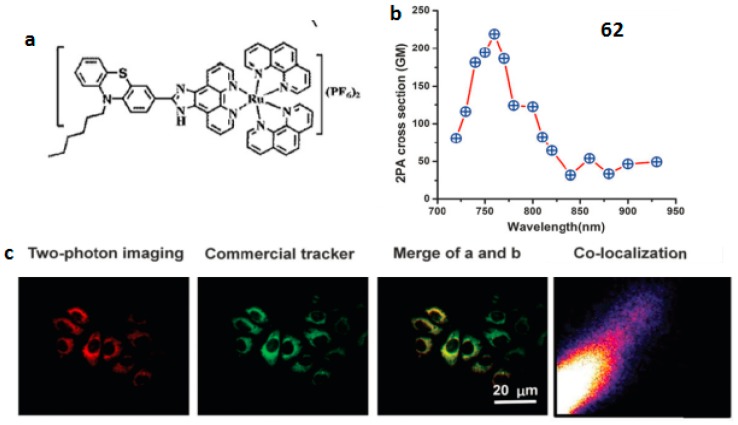
(**a**) Molecular structures of **62**; (**b**) 2PA cross-sections of **62** in DMSO (dimethylsulphoxide) solution (*c* = 1 × 10^−3^ mol·L^−1^); (**c**) 2PFM of HepG2 cells co-labeled with **52** and Mitotracker.

In 2009, Barton et al. reported a cell-penetrating peptide conjugated ruthenium complex, which could be delivered into the nucleus [136]. Thomas et al. reported a dinuclear Ru(II) polypyride complex that functions as a structure-sensitive probe for direct DNA labeling in vitro [137]. All these observations suggest that Ru(II) complexes have the potential to be used as subcellular markers. Our group designed a novel 2P fluorescent mitochondrial probe (complex HLRu (**62**) (Figure 15) [138] based on imidazo[4,5-f][1,10]phenanthroline. First, the phenothiazine group has a rich *π*-electron donating ability and high electron delocalization capability. Second, the alkyl chain can further influence the extent of electron delocalization and, consequently increase the solubility of the complex molecule. This in turn enables the use of biologically friendly solvents (e.g., PBS (phosphate buffered saline)) in later long-term live cell experiments. Remarkably, **62** displays 2PA activity in the range of 720–930 nm in DMSO (dimethylsulphoxide). The maximum 2PA cross-section *δ* for **62** is approximately 219 GM units at 760 nm, which is larger than those of the reported Ru(II) complexes [139]. The present findings, i.e., the strong near-IR 2PA response as well as the low toxicity of **62**, make it a promising material for applications in biology. The in vitro binding assay, two-photon confocal microscopy, and transmission electron microscopy elucidated that **62** enters cells via energy-dependent endocytosis and binds with mitochondrial DNA in living cells enabling tissue imaging. Due to its low cytotoxicity, large 2PA cross-section and efficient mitochondrial DNA staining, complex **62** is believed to have great potential as a biocompatible dye for mitochondrial DNA in living cells under 2PFM imaging.

**Figure 16:**
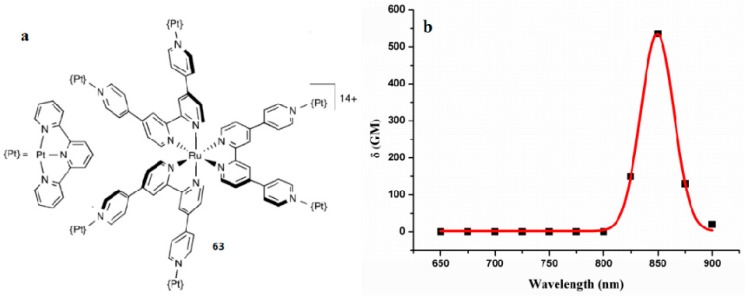
(**a**) Molecular structure of **63** (**b**) Dispersion of *δ* for **63**. The fit lines are of limited reliability due to the 25 nm separation between the data points.

Platinum complexes have attracted great interest in the past decade due to the ease of structural modification, rich photophysical properties, and versatile potential applications [140]. However, very little attention has been paid to the nonlinear optical application of the excited-state absorption of Platinum complexes. Here, we describe unusual polymetallic complexes in which the ligand 4, 4′:2′, 2″:4″, 4′′′-quaterpyridine (qpy) is used to connect either two or six Pt^II^ (tpy) units to a Ru^II^ core [140]. In consideration of the nature of the *π*-conjugated systems, the new complex **63** shows relatively large 2PA activities (Figure 16). The 2PA properties were assessed by using the Z-scan technique over the range 650–900 nm, affording the real and imaginary parts of the second hyperpolarizability *γ*, together with *δ* values. Platination causes slight redshifts and decreases the emission intensity when compared with the Ru^II^-based cores [141]. This structural change also increases the 2PA activities, which are relatively large when considering the nature of the *π*-conjugated ligand systems, up to a maximum *δ* value of 523 GM for the hexaplatinated complex **63** at 850 nm in acetone. This new complex 63 may be of interest for diverse purposes of H_2_ production and anticancer activity.

##### Transition Metal Complexes Based on Terpyridine Ligands

Polypyridyl ligands occupy a central position in modern coordination chemistry. Indeed, 2,2′-bipyridine has been described as ‘‘the most widely used ligand’’ [142]; its transition metal complexes are ubiquitous, whilst the terdentate analogue 2,2′:6′,2″-terpyridine (tpy) is not far behind [143]. Complexes of these ligands typically have high stability with respect to metal ion dissociation, owing to the synergistic combination of *σ*-donating nitrogen atoms and *π*-accepting heterocycles coupled with the favorable formation of five- and six-membered chelating rings. Moreover, the same properties help to ensure that polypyridine complexes absorb light and sometimes emit in the visible region. Typically, these metal complexes possess multiple transitions such as metal-to-ligand charge transfer (MLCT) and ligand-to-ligand charge transfer (LLCT) in the visible region. The binding of protons or cations perturbs the charge density in the binding unit and subsequently induces optical signal switching between different energy states to achieve the desired optical responses. However, it is still a challenge to design and synthesize a series of metal complexes especially transition metal complexes with low molecular weight, high water-solubility, biocompatibility, and large 2PA cross sections in the near infrared range (700–900 nm).

**Figure 17:**
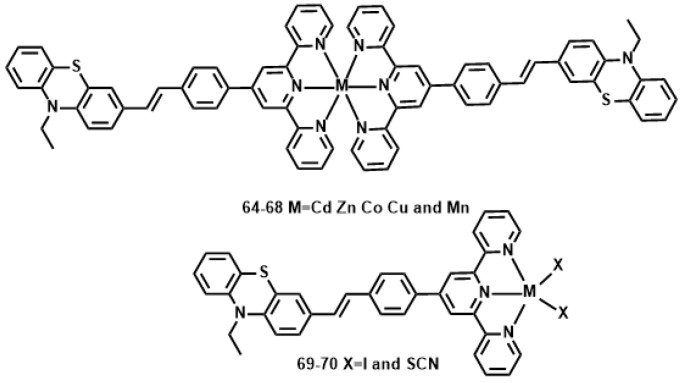
The structures of **64**–**68**.

Li et al. reported a novel 10-ethyl-3-[4-(2,2′:6′,2″-terpyridinyl-4′-yl) styryl] phenolthiazine (L) (Figure 17) [144], with the styryl moiety as the *π*-bridge; the tpy unit, tridentate ligand, can easily coordinate to various metal ions. The metal-ion binds to the acceptor, forming a D-π-A motif ligand, rather than a donor site, it should yield an increase rather than decrease of intraligand charge transfer (ICT) upon excitation. Different metal ions can influence the structures and photophysical properties of resulting complexes to a large extent, which is expected to result in an increased *δ* and enhanced fluorescence brightness. Their 2PA properties in DMF were studied using the Z-scan method. Considering the molecular weight difference between the complexes and free ligand, the 2PA cross-section values for all the complexes are approximately 2.4- to 3.6-fold larger than that for L (Table 7). Generally, the different metal centers are responsible for the different 2PA properties of the complexes. The *δ* values of the complexes **64**–**68** exhibit the order of **68** < **66** < **67** < **64** < **65**. Another factor for the larger *δ* of **65** and **66** compared to those of **66**–**68** is attributed to the closed-shell d^10^-metal centers being non-detrimental to fluorescence. Considering each complex molecule of **64**–**68** contains two ligand molecules, it is slightly surprising that **64**–**68** (with two ligands L) has such similar values to **69** and **70** (one ligand L).

**Figure 18:**
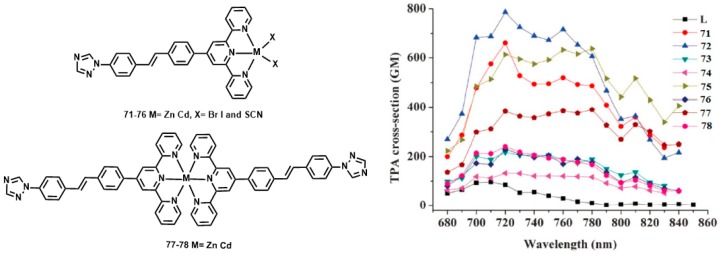
The structures of **71**–**78** and two-photon absorption cross-sections of L and its complexes **71**–**78** in DMF.

Of late, Zhou et al reported another novel D-π-A type ligand 4′-(4-[4-(1H-1,2,4-triazolyl)styryl] phenyl)-2,2′:6′,2″-terpyridine (L) and its six halogen Zn(II) and Cd(II) complexes **71**–**78** (Figure 18) [145]. The 2PEF experimental results reveal that 2PA cross-sections of these complexes are extraordinarily larger than that of the ligand, with maximum values of 97, 661, 787, 218, 133, 613, 230, 384, and 241 GM for L and complexes **71**–**78** in DMF, respectively. Density functional theory (DFT) calculations were performed on **71**–**78** and revealed that the halogen affects the accepting capability of surrounding metals due to different electron inductive effects, in the order Br > SCN > I. Thus, this in turn affects the 2PA cross-section values. The results indicate that the 2PA cross sections vary in the order Br > SCN > I, which is consistent with the experimental results.

**Figure 19:**
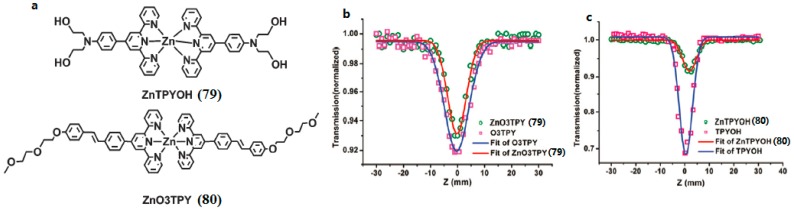
(**a**) The structures of **79**, **80**; Open-aperture Z-scan experimental data and fitting curves for **79** (**b**), **80** (**c**) and their free ligands in H_2_O–acetonitrile (4:1) under a 750 nm laser beam. The concentration was 1.0 × 10−3 M.

With the aim of obtaining terpyridines applicable for biological systems, di(hydroxyethyl) amino and (2-(2-meth-oxyethoxy)-ethoxy)benzyl groups were chosen to combine them with 2,2′:6′,2″-terpyridine. Two novel terpyridines (**79** and **80**) with good water-solubility were obtained by Shi et al. (Figure 19) [146]. All the ligands and complexes show evident two-photon absorption ability when evaluated by Z-scan technology. The two terpyridine ligands displayed good two-photon excited fluorescence (2PEF). Unlike the previous report on the enhanced 2PA ability of Cd(II), Zn(II) coordination complexes when compared with their terpyridine ligand, coordination to the Zn(II) center here led to a decrease of the 2PA response, with *δ* of 140 and 110 GM for **79** and **80**, respectively. However, the highlight of the work is that the 2PEF titration experiment revealed that **80** had a linear response to the Zn^2+^ concentration. The good water solubility and high two-photon emissive ability of **80** provide an excellent starting point for the development of cellular zinc two-photon fluorescent probes.

**Figure 20:**
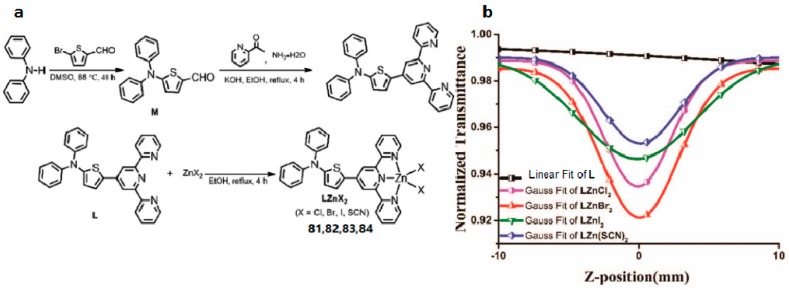
(**a**) Synthetic route for ligand L and complexes LZnCl_2_ (**81**), LZnBr_2_ (**82**), LZnI_2_ (**83**), and LZn(SCN)_2_ (**84**); (**b**) Theoretical curves of representative open-aperture Z-scan traces at wavelengths corresponding to a maximum nonlinear absorption for L, LZnCl_2_ (**81**), LZnBr_2_ (**82**), LZnI_2_ (**83**), and LZn(SCN)_2_ (**84**) at 850 nm.

It is well known that extending conjugated systems to construct a novel large D-π-A molecule contributes a positive impact on the enhancement of 2PA response. However, compounds with a large conjugated system are normally thermally unstable and their synthesis processes are time consuming [147], which limits their various practical applications. Moreover, recent studies have suggested that a large *π*-conjugated organic ligand may result in a decreasing 2PA effect on coordination with metal ions, which is contrary to the original intention [148,149,150]. Therefore, economically designing a small molecule with a D-A type if possible that possesses a stronger 2PA effect for practical applications gradually appears as a novel concept [151,152]. Considering the above, Tan et al. rationally synthesized a novel ligand 4′-(4-(diphenyl-amino)thienyl)-2,2′:6′,2″-terpyridine (L) and its Zn(II) complexes (LZnCl_2_ (**81**), LZnBr_2_ (**82**), LZnI_2_ (**83**), and LZn(SCN)_2_ (**84**)) (Figure 20) [153]. Specifically, L with D-A construction combines the electron rich properties of diphenylamine (as D) with the electron-withdrawing ability of terpyridine (as A), which can strongly bind with metal ions; Furthermore, Zn(II) within the complexes can arouse stronger charge transfer leading to strong 2PA response [154,155]. Two-photon absorption cross-section results show clearly that **81**–**84** exhibit discernible two-photon absorption values with a maximum corresponding to *δ* ≈ 2343, 2583, 907, and 1775 GM at 850 nm by using the open Z-scan method, respectively. Such a difference must be due to varied negative ions, which indicates that anions having different electronegativity may considerably influence the 2PA cross sections. The results show that the influences of electron inductive effects of different halide ions on the two-photon absorption properties (Br > Cl > SCN > I) differ from those on the linear absorption properties as mentioned previously (SCN > I > Br > Cl). This significant decrease in the two-photon absorption value of **83** could be ascribed to the heavy atom effect (HAE) of the iodine atom. Moreover, as P. N. Prasad declared [156], there was a strong correlation between intramolecular charge-transfer processes and two-photon absorptivity from the viewpoint of electronic structures and photophysical processes. This work suggested that these thiophene-based complexes be proposed as new NIR 2PA materials.

**Figure 21:**
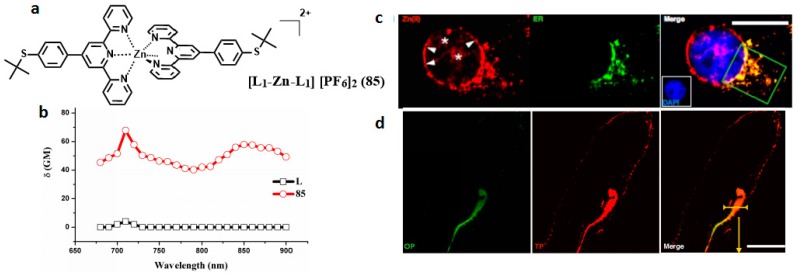
(**a**) Molecular structures of the ligands (L) and its metal complexes (**85**); (**b**) 2PA spectra of L and related complex **85**; (**c**) Two-photon image of HepG2 cells incubated with 40 μM, *λ*_ex_ = 700 nm (emission wavelength from 543 to 606 nm) and One-photon image of HepG2 cells incubated with ER tracker, *λ*_ex_ = 488 nm (emission wavelength from 500 to 550 nm); (**d**) One-photon image of 72 h-zebrafish larva incubated with 30 μM after 4 h of incubation, washed by PBS buffer, *λ*_ex_ = 405 nm (emission wavelength from 440 to 480 nm).

Because of the high toxicity of halide ions, from the ‘green’ perspective, an issue of primary importance is the solubility of products in many common organic solvents and in most green solvents including water. We demonstrated that hexafluorophosphate can be solubilized in a green aqueous solvent system—DMSO/water—in order to meet the biological requirement. Spurred by this, novel terpyridine ligands (L) and its Zn (II) complexes (**85**) (Figure 21) [157] were designed and synthesized. The complexation with Zn(II) would be likely to achieve enhanced 2PA properties due to the lack of d-d transitions in comparison with the other metal ions [158]. The 2PA cross-section values of complex **85** was 65 GM under 710 nm excitation which was significantly enhanced compared to its free ligand. Importantly, the peak 2PA cross-sections appear in the near-infrared range. This moderate 2PA response comes from the marked core to periphery charge redistribution in complexes upon photo-excitation [159]. Therefore, complexation with Zn(II) enhances the electron-acceptor character of the terpyridine group, which converts the ligand to a more strongly polarized D-A unit, which may favor ICT, causing enhanced two-photon absorption. The enhanced trend observed in the experiments was consistent with above structural investigation and theoretical calculations. Relying on the comprehensive studies of 2PA properties, **85** was selected for bioi-maging application. A bio-imaging study using two-photon scanning microscopy shows that HepG2 cells can be sufficiently stained with **85** specifically and that it is capable of specific monitoring the fluorescence signals in the intestinal system of living zebrafish larva at a depth of >100 μm. All these results favor that **85** may be a potential biocompatible and long-term in vivo and in vitro imaging agent.

**Figure 22:**
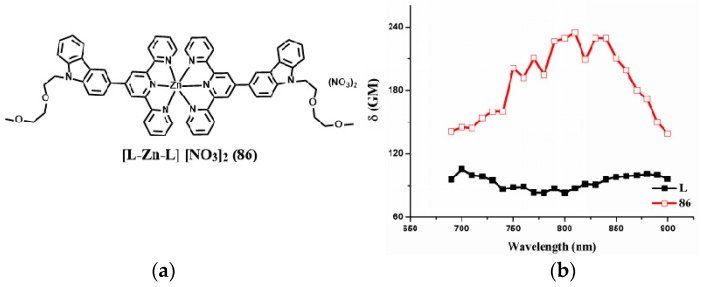
(**a**) Molecular structures of the ligand (L) and its metal complex (**86**); (**b**) 2PA spectra of L and related complex **86**.

**Figure 23:**
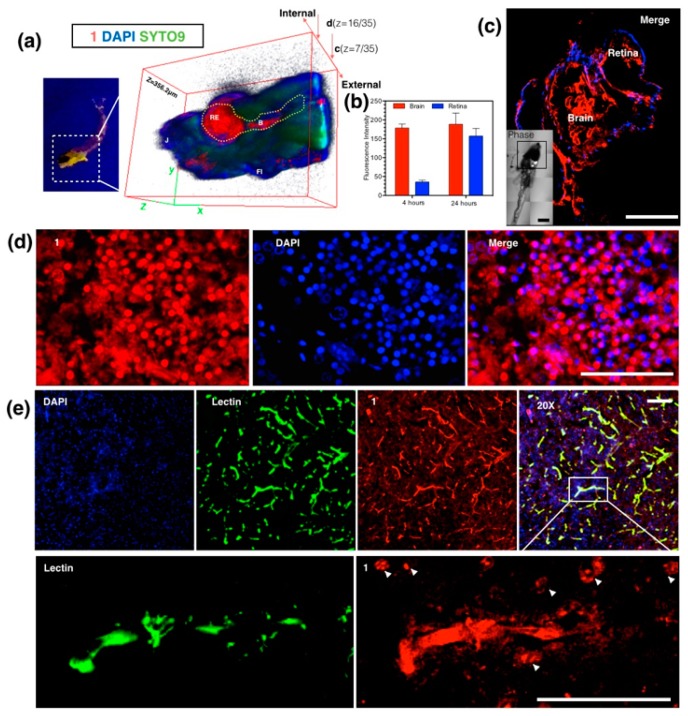
(**a**) Anaesthetized Larval zebrafish bath with **86** (100 nM) for 24 h under UV light (left) and 3D micrographs (thickness = 356.2 μm) under two-photon confocal microscopy in larval zebrafish brain and retina at 4 and 24 h; (**c**) Whole brain region imaging of staining larval zebrafish by **86** co-stained with DAPI (4,6-diamino-2-phenyl indole), insert: DIC tile scanning of the imaged larval zebrafish; (**d**) Braco-stained with DAPI, Syto9 and Propidium Iodide; (**b**) Fluorescence intensity analysis of uptake of **86** in sections of staining adult zebrafish by **86** co-stained with DAPI; (**e**) Ex vivo assessment of **86** following i.v. injection in mice co-labeled with DAPI and Alexa488-lectin, and zoom in (200×) micrographs show the detail of brain capillaries imaged by confocal laser scanning microscopy, with strong internalization of **86** with brain endothelium and CNS (central nervous system) cells at nuclear and plasma membrane. The scale bar represents 200 μm. Error bars: SEM (scanning electron microscope), *n* = 3. Abbreviations: Re = retina, B = brain, J = jaw, FI = fin.

In order to further increase the terpyridine complexes’ solubility especially those that can be completely dissolve in water, Tian et al. fabricated a novel D-A type complex (**86**) in which the terpyridine ring acts as tridentate chelate group and a carbazole moiety as a two-photon active moiety. Extended polyether chain and nitrate serves as a steric hindrance to diminish the intermolecular quenching effect, as well as good water solubility; it was also widely applied in an in vivo drug delivery system due to their immune ‘stealth-like’ behavior leading to increased blood circulation time and lowed toxicity (Figure 22) [160]. Coordinating with Zn(II) of the designed ligand would be likely to lead to enhanced 2PA response and fluorescence brightness due to the lack of d-d transitions. It is proved once again that an extended conjugation bridge, for instance in the *σ*-bridge system, may favor ICT and thus cause significantly enhanced two-photon absorption. Intriguingly, **86** can be used as an imaging probe to simultaneously light up plasma membrane and nucleus in living cells via an endocytotic pathway. Effective diffusion in the brain compartment in larval and adult zebrafish was observed over a depth of ~300 μm (Figure 23). Particularly, in a mouse model, intravenously administered **86** displayed high affinity with BBB (Blood Brain Barrier) and showed good penetration into brain parenchymal. In fact, images obtained in this study, especially those in mammalian tissues, we are enthusiastic that, in the future, class-specific small molecular probes based on the terpyridine group with further modification/functionalization will be discovered systematically and comprehensively.

##### Transition Metal Complexes Based on Bis-*β*-Diketonate

**Figure 24:**
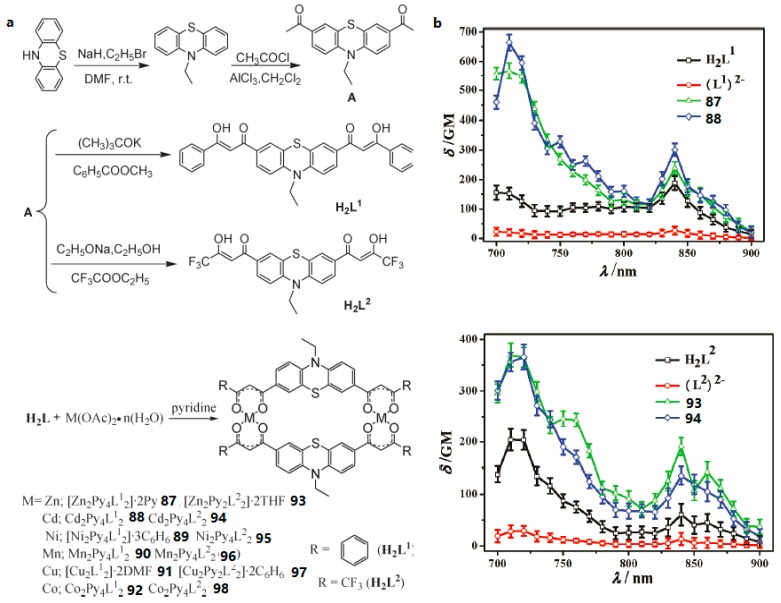
(**a**) Synthesis of two bis-*β*-diketones H_2_L^1^, H_2_L^2^ and their complexes **87**–**98**; (**b**) 2PA spectra of all the compounds (*c* = 0.1 mM) in THF.

**Figure 25:**
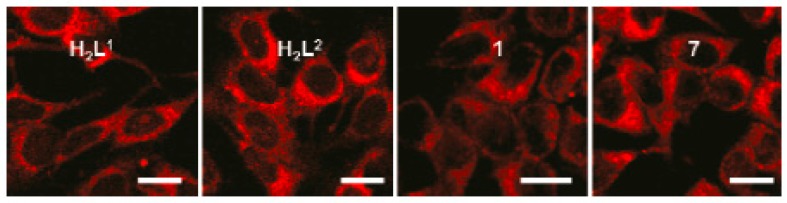
Fluorescent imaging of two-photon microscopy of MCF-7 cells, *λ*_ex_ = 720 nm, from left to right: H_2_L^1^, H_2_L^2^, **87**, and **93** (all the scale bars represent 10 μm).

*β*-diketonate derivatives can act as good bidentate ligands, after losing a proton, coordinating to metal ions to form a *π*-conjugated unit. Therefore, *β*-diketone ligands can form a higher degree of *π*-electron delocalization in the metal complexes due to an extended *π*-bridge by *π*-electron contribution from the metal ions. Significantly, their metal complexes should possess excellent 2PA properties. On the basis of our interest in searching for optimized molecular structures having large molecular 2PA cross sections, Li et al. constructed two novel ligands based on phenothiazine with *β*-diketone units (H_2_L^1^, H_2_L^2^) and their transition metal complexes (Ni(II), Mn(II), Co(II), Zn(II), Cd(II) **87**–**98**) (Figure 24) [161]. The *δ* values for H_2_L^1^ and H_2_L^2^ are ca. 155 (700 nm) and 204 GM (710 nm), respectively, which is in line with the electron-accepting ability of the groups: CF_3_ > C_6_H_6_. Considering each complex molecule containing two ligand molecules, *δ*_max_ values of **87** and **88** are ca. 568 and 664 GM at 710 nm, 14- and 16-fold larger than that of the real ligand (L^1^)^2−^. Also, the peak *δ* values for **93** and **94** are ca. 369 (710 nm) and 366 GM (720 nm), 7-fold larger than that of the real ligand (L^2^)^2−^. Thus, dramatically increased two photon fluorescence can be achieved by using the closed-shell d^10^ metal centers such as Zn^2+^ and Cd^2+^. As Zn has relatively low toxicity towards live cells, two-photon microscopy of MCF-7 cells labeled with **87** and **93** were captured, respectively. The two-photon microscopy show that **87** and **93** are presented within the cells (*λ*_ex_ = 720 nm) (Figure 25). Herein, the cytoplasmic distribution is more uniform. A few bright spots are observed in the nucleus indicating that the compounds may target small organelles in the nuclear region called nucleoli. However, corresponding light-field images of the cells ascertaining the locations of the cell nuclei and nucleoli, and a co-staining experiment to confirm the nucleoli-staining capability of the ligands and complexes will be carried out in the future. It is showing the remarkable stability in cellular cytosol as we switch from one-photon excitation to two-photon excitation. Those complexes might have potential as safety fluorescence probes which coud be avoided photon-damage effectively. This work paves the way for the designation of either novel types of bis-diketones, their complexes or new effective chromophores with two-photon activity for TPM images of live cells.

#### 3.3.3. Clusters

**Figure 26:**
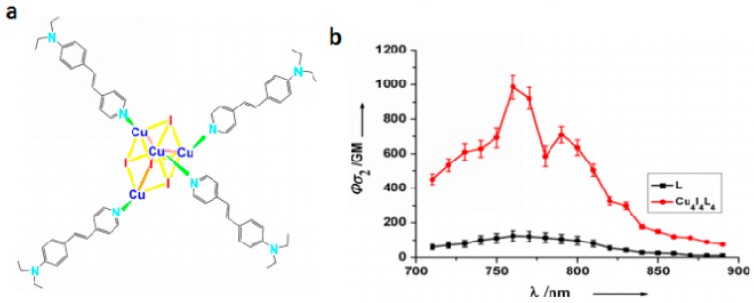
(**a**) Molecular structures of Cu_4_I_4_L_4_ (**99**); (**b**) Two-photon absorption action spectra of L and Cu_4_I_4_L_4_ (**99**) in ethanol (*c* = 1.0 × 10^−4^ mol·L^−1^).

**Figure 27:**
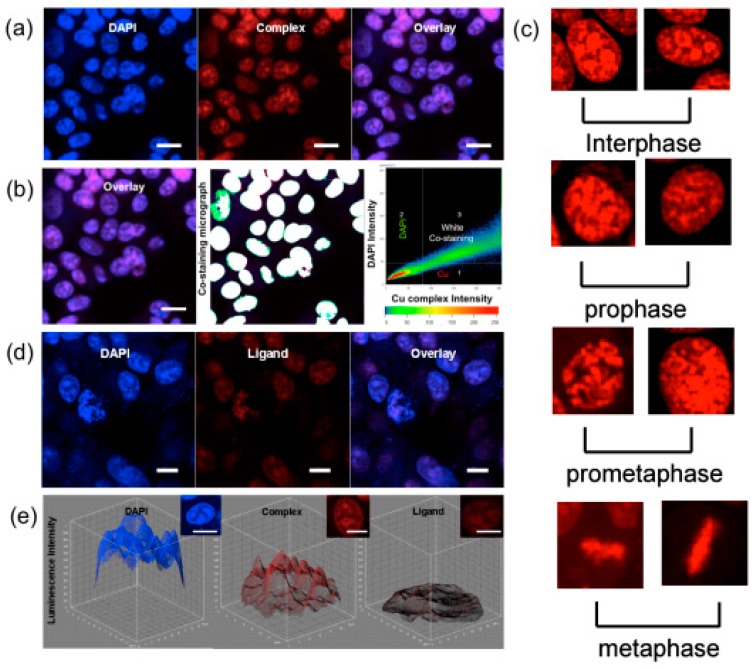
2PFM of MCF-7 at: (**a**) Containing Cu_4_I_4_L_4_ (**99**) (mid red) with DAPI (left blue) and overlay image (right); (**b**) Co-localization micrograph profile prove that the co-staining region corresponding to the white colocalization part which is located in the nuclear section; (**c**) Cell chromosome through progression of m-phase shows cellular luminescence by uptake cluster; (**d**) Containing L (mid red) with DPAI (left blue) and overlay image; (**e**) 3D intensity profile of DAPI, Cu_4_I_4_L_4_ (**99**) (200 μM) and L (200 μM) luminescence across a MCF-7 single cell after incubation for 2 h. (All scale bars represent 10 μM.)

Another exciting example of the use of phosphorescent heavy-metal complexes for two-photon bio-imaging are the coordination clusters because they involve dπ-pπ delocalized systems and dπ-dπ conjugated systems [162]. These clusters have a large variety of structures and diverse electronic properties that can be tuned by virtue of the coordinated metal ions. The clusters can also extend the electronic delocalization, which should increase their *δ* values. We have paid attention to enhance the *δ* value by combining functional ligands with metal clusters. Copper(I) iodide (CuI) clusters can be used as precursors to react with the bulky ancillary organic ligands, which are favorable for the formation of multi-branched clusters with large *δ* in the near-IR region. This is most likely due to the following three facts: (i) the closed-shell d^10^ Cu(I) shows a variety of coordination formats; (ii) CuI shows higher energy emission bands than its polymorphs (CuBr, CuCl) [163], and can be facilely modified to form various structures; (iii) the multi-branched cluster can extend electronic delocalization and polarity of rigid conjugated structural motifs (organic ligand), which would favor intra/intermolecular charge transfer and thus help to enhance 2PA. With the above considerations, we designed a novel multi-branched cuprous cluster (Cu_4_I_4_L_4_ (**99**), L = (E)-(4-diethylanilino-styryl)pyridine, Figure 26) [164] possessing larger conjugation motifs to enhance 2PA for a brighter 2PFM. Figure 27 indicates that in the measured range, the 2PA cross-sections *δ* of **99** were enhanced with increasing polarity compared to its free ligand, especially in ethanol. This large 2PA response comes from the marked core to periphery charge redistribution in **99** upon photoexcitation [165]. Therefore, complexation with Cu(I) enhances the electron-acceptor character of the central pyridine group, which converts the ligand to a more strongly polarized D-π-A unit, making this cluster a potential candidate for 2PA responses. It should be noted that this *δ* value exceeds that of many fluorophores widely used in biology, including fluorescein, BODIPY, DAPI, GFP, or other complexes [166,167,168]. Significantly, compared with those with free L, not only **99** bears larger *δ* values, but also the cluster exhibits higher quantum yield and longer lifetime in ethanol, which spurred us further to explore its potential application in biological imaging.

The 2PFM images (Figure 25) of each live cell were successfully taken and clearly display the nucleus structure. To determine the location of the luminescence, a traditional nuclear dye 4′,6-diamidino-2-phenylindole (DAPI) was also used. Figure 16a,b show that the cluster molecules have been taken up by cellular nuclear, Figure 16c reveals the TPA luminescence from chromosomes of cells which are both undergoing inter phase and mitotic phases. Control experiments for **99** and DAPI image (Figure 25a) indicate there is no connection between **99** and DAPI. Hence, **99** is clearly capable of detecting the nuclear section in MCF-7 cells. The excitation and emission wavelengths of **99** probes are 780–800 nm and 685 nm, respectively, which can be attributed to excited-state equilibrium via intersystem crossing (ISC) and triplet energy transfer (TET) [169], and are different from that of the free ligand. Similar nuclear stain properties shown by L using the same concentration (Figure 25d), however, show luminescence intensity to drop approximately 1/5 times lower than Cu_4_I_4_L_4_ (**99**) (Figure 25e).

**Figure 28:**
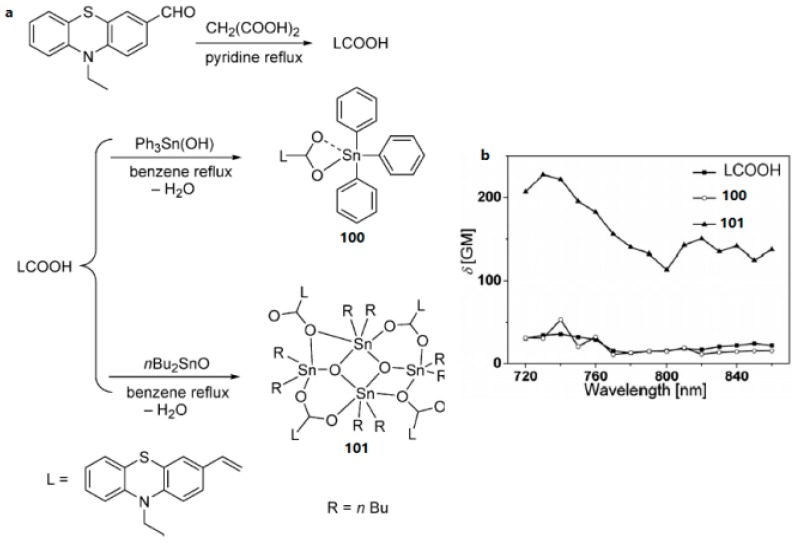
(**a**) Synthesis of the organotin carboxylate derivatives LCOOH, **100**, and **101**; (**b**) 2PA spectra of LCOOH, **100**, and **101**.

Research on organometallic compounds has aroused great interest in recent years. Their intimate connection is demonstrated with the synthesis and assembly of new materials, excellent optical properties, catalysis reactions, and exploitation of many anti-cancer drugs etc. [170,171,172,173], wherein the organotin compounds, especially ubiquitous organotin carboxylates, exhibit a range of effects for chemotherapy, biological, catalytic and industrial applications [174,175,176,177]. Although their properties including diversity of structures for constructing novel compounds, assembly/self-assembly for polymers, and anti-tumor/anti-bacterial have been systematically investigated [178,179,180,181], only a few studies regarding the two-photon absorption properties of the organotin carboxylate derivatives have been reported. Hence, our efforts have been directed towards the search for optimized molecular structures having large 2PA cross-sections. In 2009, Li et al. reported a (*E*)-3-(10-ethylphenothia-zine-3-yl)-acrylic acid (LCOOH) which reacted with Ph_3_Sn(OH) and nBu_2_SnO to afford the mono- and tetranuclear products (**100** and **101**) (Figure 28) [182], respectively. The tetranuclear product **101** was obtained in a nearly quantitative yield, with four LCOO– units around the organooxotin cluster through the stannoxane-based one-step synthetic strategy. It is obvious that **101** shows the best 2PA property and the *δ* value of **101** is much higher than that of the ligand. This is mainly attributed to the increase in the number of LCOO– units in **101**. By considering the molecular weight difference between the complexes and the metal-free chromophores, the *δ* values for **100** and **101** were calculated to be ca. 1.5-times larger than that for LCOOH.

**Figure 29:**
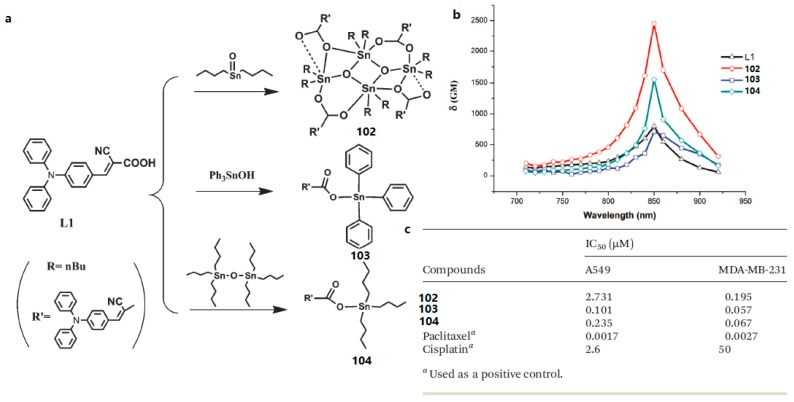
(**a**) Synthetic routes for L1 and complexes **102**, **103**, **104**; (**b**) 2PA cross-sections of L1 and complexes **102**–**104** in CH_2_Cl_2_ solution; (**c**) Inhibitory concentration IC_50_ (μM) of **102**–**104** against tumor cell lines.

Based on Li’s work, the other three novel organooxotin complexes (**102**, **103**, and **104**) were synthesized by reaction of L1 (2-cyano-3-(4-(diphenylamino)phenyl) acrylic acid) with *n*-Bu_2_SnO, Ph_3_Sn(OH) and *n*-Bu_6_Sn_2_O by Zhao et al. (Figure 29) [183]. The 2PEF of the complexes have been systematically studied, suggesting that the three metal complexes have strong two-photon absorption (2PA) and large 2PA cross-sections *δ*. The maximum *δ* are 810 GM for the ligand, 2500 GM for **102**, 780 GM for **103**, 1600 GM for **104** in CH_2_Cl_2_ solution. It is noteworthy that **104** exhibits larger 2PA cross-section per molecular weight compared to the ligand. Finally, high anti-tumor activity of these three metal complexes has also been identified. The capability of the three organotin complexes as potential anti-tumor agents were preliminary tested in vitro against A549 and MDA-MB-231 cell lines. The results of the cytotoxic effects (inhibitory concentration IC_50_) of complexes **102**, **103**, and **104** against the two tumor cell lines were compared using paclitaxel and cis-platin as positive controls. Although the IC_50_ values of three complexes are relatively lower than paclitaxel against two tumor cell lines, **102** and **103** exhibited much more anti-tumor activity than cis-platin (25.7 and 11.1-fold lower IC_50_ for Z2 and Z3 respectively in A549 cells, 877.2 and 746.3-fold respectively in MDA-MB-231 cells). In particular, among these three complexes, **103** is a relatively small molecule and the most efficient anti-tumor agent for the A549 and MDA-MB-231 cell lines, so it can be used as a potential anti-tumor agent. This study paves the way to design organotin compounds with large two-photon absorptions and good antitumor effects.

## 4. Conclusions and Outlook

In this review, we summarized our recent works in 2PA materials based on organic, inorganic, organic-inorganic hybrid, and complexes system. These 2PA materials exhibit systematic 2PA properties and well-defined cell uptake behavior, allowing the use of two-photon microscopy to track their fate in cellulo as well as small-animal bio-imaging. Considering the advantages and disadvantages of the present 2PA materials as bio-imaging materials, several directions in this field in the future should be followed in order to develop high quality 2PA bio-imaging materials and exploit their further applications:

(1) For sensing, diagnostic or therapeutic applications, it is very important to realize the rapid and efficient cellular uptake of 2PA materials by optimizing their overall charge, size, hydrophobicity, and conjugated moiety.
(2) Although a series of 2PA materials have been developed to stain different cellular organisms, their compartmentalization staining is usually not specific. Therefore, selective compartmentalization staining is still a challenge.
(3) Compared with rapid developments in the synthesis of 2PA materials as staining dyes, discussions on the internalization mechanisms of phosphorescent complexes are still rare.
(4) Compared with two-photon rare earth complexes, the long lifetime of phosphorescence can allow the possibility of two-photon time-resolved emission imaging. As a result, we have the emissive signals of the two-photon rare earth complexes using two-photon time-resolved emission imaging microscopy. However, the examples of two-photon lifetime-based imaging are very few. This will be another important research direction.
(5) Compared with the rapid development of two-photon bio-imaging based on 2PA materials, only a few complexes have been used in multi-model bio-imaging, such as, Magnetic Resonance Imaging (MRI) combined with two-photon fluorescence microscopy (TPFM). Therefore, realizing multi-model imaging will be an important research topic in this field.

In summary, the rich choice of 2PA materials produces rich and tunable two-photon fluorescence properties. Hence, 2PA materials have become a class of very attractive bio-imaging materials. It is expected that more and more excellent 2PA bio-imaging materials will be developed in the years to come.

## Figures and Tables

**Table 1:** Photophysical data of **1**–**10**.

Compounds	λ_max_^a^ (nm)	λ_max_^b^ (nm)	λ_max_^c^ (nm)	λ_max_^d^ (nm)	*δ* ^e^
**1**	471	603	621	1064	134
**2**	482	601	627	1064	122
**3**	486	604	627	1064	136
**4**	486	605	625	1064	123
**5**	426	521	586	760	14.8
**6**	340	585	615	750	309
**7**	480	603	598	960	48
**8**	485	606	598	960	57
**9**	474	597	600	960	115
**10**	472	595	620	960	517

^a^ Absorption peak position in nm (1 × 10^−5^ mol·L^−1^); ^b^ Peak position of SPEF (Single Photon Excited Fluorescence) in nm (1.0 × 10^−5^ mol·L^−1^), excited at the absorption maximum; ^c^ 2PEF (Two-Photon Excited Fluorescence) peak position in nm pumped by femtosecond laser pulses at their maximum excitation wavelength; ^d^ 2PA (Two-Photon Absorption) maximum excitation wavelength; ^e^ 2PA cross section in GM Göppert-Mayer units (1GM = 10^−50^ cm^4^s·photons^−1^·molecule^−1^). Solvent: DMF (Dimethyl Formamide).

**Table 2:** Photophysical data of **11**–**21**.

Compounds	λ_max_^a^ (nm)	λ_max_^b^ (nm)	λ_max_^c^ (nm)	λ_max_^d^ (nm)	*δ* ^e^
**11**	380	457	495	720	8
**12**	402	443	483	740	10
**13**	452	566	572	820	498
**14**	406	520	570	800	104
**15**	416	529	550	870	151
**16**	471	573	600	830	1319
**17**	460	569	590	800	1885
**18**	480	630	610	820	375
**19**	451	600	590	840	216
**20**	458	561	560	840	742
**21**	459	561	562	840	170

^a^ Absorption peak position in nm (1 × 10^−5^ mol·L^−1^); ^b^ Peak position of SPEF in nm (1.0 × 10^−5^ mol·L^−1^), excited at the absorption maximum; ^c^ 2PEF peak position in nm pumped by femtosecond laser pulses at their maximum excitation wavelength; ^d^ 2PA maximum excitation wavelength; ^e^ 2PA cross section in GM. Solvent: DMF.

**Table 3:** Photophysical data of **22**–**49**.

Compounds	λ_max_^a^ (nm)	λ_max_^b^ (nm)	λ_max_^c^ (nm)	λ_max_^d^ (nm)	*δ* ^e^
**22**	286, 357	487	525	720	434
**23**	276, 327, 406	-	-	760 (Z-scan)	415 (Z-scan)
**24**	276, 327, 409	-	-	760 (Z-scan)	462 (Z-scan)
**25**	277, 352	446	495	720	179
**26**	286, 369	526	575	750	70
**27**	285, 370	485	580	750	295
**28**	285, 375	487	574	750	308
**29**	338, 429	494	-	690 (Z-scan)	7938 (Z-scan)
**30**	294, 526	526	562	720	2869
**31**	290, 360	505	520	840	1019
**32**	370	470	500	690	327
**33**	412	500	550	700	394
**34**	347, 438	570	605	940	660
**35**	349, 449	583	608	940	999
**36**	348, 484	591	613	860	1830
**37**	350, 497	602	620	880	2087
**38**	355, 484	596	623	860	5382
**39**	355, 499	602	629	860	9398
**40**	295, 410	548	566	890	121
**41**	297, 442	569	603	890	138
**42**	374	417	442	720	220
**43**	374	478	528	700	777
**44**	396	517	536	780	623
**45**	387	498	527	780	595
**46**	387	495	510	780	285
**47**	398	509	522	780	392
**48**	397	505	530	780	287
**49**	388	503	523	780	190

^a^ Absorption peak position in nm (1 × 10^−5^ mol·L^−1^); ^b^ Peak position of SPEF in nm (1.0 × 10^−5^ mol·L^−1^), excited at the absorption maximum; ^c^ 2PEF peak position in nm pumped by femtosecond laser pulses at their maximum excitation wavelength; ^d^ 2PA maximum excitation wavelength; ^e^ 2PA cross section in GM. Solvent: DMF.

**Table 4:** Open- and closed-aperture Z-scan measurement data for the third-order nonlinearity parameters of S, S-Au NPs (**51**) in DMF with 10^−3^ M concentration.

Non-linearity Parameters	S	S-Au NPs	Au NPs
*λ*_max_^a^ (nm)	790	790	790
*β* (cm·GW^−1^)	0.11	0.14	0.069
*δ* (GM)	4595	5849	2874
*γ* (cm^2^·GW^−1^) (×10^−14^)	4.62	5.19	-
Re(*χ*^(3)^) [esu] (×10^−14^)	2.40	2.69	-
Im(*χ*^(3)^) [esu] (×10^−16^)	3.59	4.60	-

^a^ Best nonlinear absorption wavelength.

**Table 5:** Open- and closed-aperture Z-scan measurement data for the nonlinearity parameters of L nanorods and the nanohybrid **53**.

Non-Linearity Parameters	L Nanorods	Nanohybrid 53
*λ*_max_^a^ (nm)	800	840
*β* (cm·GW^−1^)	0.58	1.60
*δ* (GM)	1353	5731
*γ* (cm^2^·GW^−1^)	5.81 × 10^−16^	1.10 × 10^−14^
Re(*χ*^(3)^) [esu]	3.01 × 10^−14^	5.70 × 10^−13^
Im(*χ*^(3)^) [esu]	1.07 × 10^−7^	5.07 × 10^−7^

^a^ Best nonlinear absorption wavelength.

**Table 6:** Open- and closed-aperture Z-scan measurement data for the third-order nonlinear parameters of **57**–**61**.

Non-linearity Parameters	57	58	59	60	61
*λ*_max_^a^	730	730	740	730	730
*β* (cm·GW^−1^)	0.086	0.105	0.032	0.049	0.083
*δ* (GM)	3888	4747	1427	2228	3766
*γ* (cm^2^·GW^−1^) (×10^−15^)	8.12	5.02	4.30	5.25	7.09
Re(*χ*^(3)^) [esu] × 10^−13^	4.51	2.79	2.39	2.92	3.94
Im(*χ*^(3)^) [esu] × 10^−14^	2.78	3.39	1.05	1.59	2.69

^a^ Best nonlinear absorption wavelength.

**Table 7:** Open-aperture Z-Scan measurement data for the third-order nonlinear parameters of **64**–**70** in DMF.

Compound	*λ*_max_^a^	*λ*_max_^b^	*β* × 10^−3^ (cm·GW^−1^)	*δ* × 10^3^ GM
L	306,394	491,561	14	5.8
**64**	310,396	492,564	49	20
**65**	315,397	494,562	51	21
**66**	326,401	496,564	45	19
**67**	308,396	499,572	38	15
**68**	311,397	495,572	34	14
**69**	320,301	491,571	47	19
**70**	320,400	491,570	43	18

**^a^** Linear absorption maxima (nm); **^b^** Linear emission maxima (in nm, under the excitation wavelength of 310 nm).

## References

[1] Goppert-Mayer M. (1931). Two-quantum processes. Ann. Phys..

[2] Maiman T. (1960). NPG library. Nature.

[3] Kaiser W., Garrett C.G.B. (1961). Two-photon excitation in CaF_2_:Eu^2+^. Phys. Rev. Lett..

[4] So B.K., Lee K.S., Lee S.M., Lee M.K., Lim T.K. (2003). Synthesis and linear/nonlinear optical properties of new polyamides with DANS chromophore and silphenylene groups. Opt. Mater..

[5] Kim H.M., Cho B.R. (2015). Small-molecule two-photon probes for bio-imaging applications. Chem. Rev..

[6] Park Y.I., Lee K.T., Suh Y.D., Hyeon T. (2015). Upconverting nanoparticles: A versatile platform for wide-field two-photon microscopy and multi-modal in vivo imaging. Chem. Soc. Rev..

[7] Niu W.F., Guo L., Li Y.H., Shuang S.M., Dong C., Wong M.S. (2016). Highly selective two-photon fluorescent for ratiometric sensing and imaging cysteine in mitochondria. Anal. Chem..

[8] Li J., Cheng F., Huang H., Li L., Zhu J.J. (2015). Nanomaterial-based activatable imaging probes: From design to biological applications. Chem. Soc. Rev..

[9] Tang J., Cai Y.B., Jing J., Zhang J.L. (2015). Unravelling the correlation between metal induced aggregation and cellular uptake/subcellular localization of Znsalen: An overlooked rule for design of luminescent metal probes. Chem. Sci..

[10] Ma D.L., He H.Z., Leung K.H., Chan D.S.H., Leung C.H. (2013). Bioactive Luminescent Transition-Metal Complexes for Biomedical Applications. Angew. Chem. Int. Ed..

[11] Qian L., Li L., Yao S.Q. (2016). Two-Photon Small Molecule Enzymatic Probes. Acc. Chem. Res..

[12] Yuan L., Wang L., Agrawalla B.K., Park S.J., Zhu H., Sivaraman B., Chang Y.T. (2015). Development of targetable two-photon fluorescent probes to image hypochlorous acid in mitochondria and lysosome in live cell and inflamed mouse model. J. Am. Chem. Soc..

[13] Griesbeck S., Zhang Z., Gutmann M., Lühmann T., Edkins R.M., Clermont G., Blanchard-Desce M. (2016). Water-soluble triarylborane chromophores for one-and two-photon excited fluorescence imaging of mitochondria in cells. Chem. Eur. J..

[14] LaFratta C.N., Fourkas J.T., Baldacchini T., Farrer R.A. (2007). Mehrphotonen-Mikrofabrikation. Angew. Chem. Int. Ed..

[15] LaFratta C.N., Fourkas J.T., Baldacchini T., Farrer R.A. (2007). Multiphoton fabrication. Angew. Chem. Int. Ed..

[16] Teran N.B., He G.S., Baev A., Shi Y., Swihart M.T., Prasad P.N., Reynolds J.R. (2016). Twisted Thiophene-Based Chromophores with Enhanced Intramolecular Charge Transfer for Cooperative Amplification of Third-Order Optical Nonlinearity. J. Am. Chem. Soc..

[17] Yu J., Cui Y., Xu H., Yang Y., Wang Z., Chen B., Qian G. (2013). Confinement of pyridinium hemicyanine dye within an anionic metal-organic framework for two-photon-pumped lasing. Nat. Commun..

[18] Yu J., Cui Y., Wu C.D., Yang Y., Chen B., Qian G. (2015). Two-photon responsive metal-organic framework. J. Am. Chem. Soc..

[19] Huang B. (2015). Native Point Defects in CaS: Focus on Intrinsic Defects and Rare Earth Ion Dopant Levels for Up-converted Persistent Luminescence. Inorg. Chem..

[20] Lin T.C., Chung S.J., Kim K.S., Wang X., He G.S., Swiatkiewicz J., Prasad P.N. (2003). Organics and polymers with high two-photon activities and their applications. Adv. Polym. Sci..

[21] Lu K., He C., Lin W. (2015). A chlorin-based nanoscale metal-organic framework for photodynamic therapy of colon cancers. J. Am. Chem. Soc..

[22] Zeng C., Shang W., Liang X., Liang X., Chen Q., Chi C., Tian J. (2016). Cancer Diagnosis and Imaging-guided Photothermal Therapy Using a Dual-modality Nanoparticle. ACS Appl. Mater. Interfaces.

[23] Chen G., Roy I., Yang C., Prasad P.N. (2016). Nanochemistry and Nanomedicine for Nanoparticle-based Diagnostics and Therapy. Chem. Rev..

[24] Liu Z.Q., Fang Q., Wang D., Xue G., Yu W.T., Shao Z.S., Jiang M.H. (2002). Trivalent boron as acceptor in D-π-A chromophores: Synthesis, structure and fluorescence following single-and two-photon excitation. Chem. Commun..

[25] Collings J.C., Poon S.Y., Droumaguet C.L., Charlot M., Katan C., Pålsson L.-O., Beeby A., Mosely J.A., Kaiser H.M., Kaufmann D. (2009). The Synthesis and one-and two-photon optical properties of dipolar, quadrupolar and octupolar donor–acceptor molecules containing dimesitylboryl groups. Chem. Eur. J..

[26] Charlot M., Porrès L., Christopher D., Entwistle C.C., Beeby A., Marder T.B., Blanchard-Desce M. (2005). Investigation of two-photon absorption behavior in symmetrical acceptor–π–acceptor derivatives with dimesitylboryl end-groups. Evidence of new engineering routes for TPA/transparency trade-off optimization. Phys. Chem. Chem. Phys..

[27] Liu Z.Q., Fang Q., Wang D., Cao D.X., Xue G., Yu W.T., Lei H. (2003). Trivalent boron as an acceptor in donor-π-acceptor-type compounds for single-and two-photon excited fluorescence. Chem. Eur. J..

[28] Liu Z.Q., Fang Q., Cao D.X., Wang D., Xu G.B. (2004). Triaryl boron-based A-π-A vs triaryl nitrogen-based D-π-D quadrupolar compounds for single-and two-photon excited fluorescence. Org. Lett..

[29] Liu Z., Shi M., Li F., Fang Q., Chen Z., Yi T., Huang C. (2005). Highly selective two-photon chemosensors for fluoride derived from organic boranes. Org. Lett..

[30] Ji L., Fang Q., Yuan M., Liu Z., Shen Y., Chen H. (2010). Switching high two-photon efficiency: From 3,8,13-substituted triindole derivatives to their 2,7,12-Isomers. Org. Lett..

[31] Chen P., Marshall A.S., Chi H.S., Yin X., Perry J.W., Jäkle F. (2015). Luminescent quadrupolar borazine oligomers: Synthesis, photophysics, and two-photon absorption properties. Chem. Eur. J..

[32] Makarov N.S., Mukhopadhyay S., Yesudas K., Brédas J.L., Perry J.W., Pron A., Mullen K. (2012). Impact of electronic coupling, symmetry, and planarization on one-and two-photon properties of triarylamines with one, two, or three diarylboryl acceptors. J. Phys. Chem. A..

[33] Ji L., Edkins R.M., Sewell L.J., Beeby A., Batsanov A.S., Fucke K., Boucekkine A. (2014). Experimental and theoretical studies of quadrupolar oligothiophene-cored chromophores containing dimesitylboryl moieties as π-accepting end-groups: Syntheses, structures, fluorescence, and one-and two-photon absorption. Chem. Eur. J..

[34] Entwistle C.D., Collings J.C., Steffen A., Pålsson L.O., Beeby A., Albesa-Jové D., Poon S.Y. (2009). Syntheses, structures, two-photon absorption cross-sections and computed second hyperpolarisabilities of quadrupolar A-π-A systems containing E-dimesitylborylethenyl acceptors. J. Mater. Chem..

[35] Chen G., Qiu H., Prasad P.N., Chen X. (2014). Upconversion nanoparticles: Design, nanochemistry, and applications in theranostics. Chem. Rev..

[36] Xu G., Zeng S., Zhang B., Swihart M.T., Yong K.T., Prasad P.N. (2016). New Generation Cadmium-Free Quantum Dots for Biophotonics and Nanomedicine. Chem. Rev..

[37] Carrone G., Etchenique R. (2015). Chemical two-photon fluorescence. Anal. Chem..

[38] Chan K.A., Kazarian S.G. (2016). Attenuated total reflection Fourier-transform infrared (ATR-FTIR) imaging of tissues and live cells. Chem. Soc. Rev..

[39] Pawlicki M., Collins H.A., Denning R.G., Anderson H.L. (2009). Two-photon absorption and the design of two-photon dyes. Angew. Chem. Int. Ed..

[40] Goodson T.G. (2005). Optical excitations in organic dendrimers investigated by time-resolved and nonlinear optical spectroscopy. Acc. Chem. Res..

[41] Sheik-Bahae M., Said A.A., Wei T.H., Hagan D.J., Van Stryland E.W. (1990). Sensitive measurement of optical nonlinearities using a single beam. IEEE J. Quantum Electron..

[42] Kamada K., Matsunaga K., Yoshino A., Ohta K. (2003). Two-photon-absorption-induced accumulated thermal effect on femtosecond Z-scan experiments studied with time-resolved thermal-lens spectrometry and its simulation. J. Opt. Soc. Am. B.

[43] Xu C., Webb W.W. (1996). Measurement of two-photon excitation cross sections of molecular fluorophores with data from 690 to 1050 nm. J. Opt. Soc. Am. B.

[44] Drobizhev M., Stepanenko Y., Dzenis Y., Karotki A., Rebane A., Taylor P.N., Anderson H.L. (2005). Extremely strong near-IR two-photon absorption in conjugated porphyrin dimers: Quantitative description with three-essential-states model. J. Phys. Chem. B.

[45] Makarov N.S., Drobizhev M., Rebane A. (2008). Two-photon absorption standards in the 550–1600 nm excitation wavelength range. Opt. Express.

[46] Wurth C., Pauli J., Lochmann C., Spieles M., Resch-Genger U. (2012). Integrating sphere setup for the traceable measurement of absolute photoluminescence quantum yields in the near infrared. Anal. Chem..

[47] Denk W., Strickler J.H., Webb W.W. (1990). Two-photon laser scanning fluorescence microscopy. Science.

[48] Rumi M., Ehrlich J.E., Heikal A.A., Perry J.W., Barlow S., Hu Z., Mc Cord-Maughon D., Parker T.C., Röckel H., Thayu-manavan S. (2000). Structure-Property Relationships for Two-Photon Absorbing Chromophores: Bis-Donor Diphenylpolyene and Bis(styryl)benzene Derivatives. J. Am. Chem. Soc..

[49] Signorini R., Ferrante C., Pedron D., Zerbetto M., Cecchetto E., Slaviero M., Fortunati I., Collini E., Bozio R., Abbotto A. (2008). Effective Two-Photon Absorption Cross Section of Heteroaromatic Quadrupolar Dyes: Dependence on Measurement Technique and Laser Pulse Characteristics. J. Phys. Chem. A.

[50] Padilha L.A., Webster S., Hu H., Przhonska O.V., Hagan D.J., Van Stryland E.W., Bondar M.V., Davydenko I.G., Slominsky Y.L., Kachkovski A.D. (2008). Excited state absorption and decay kinetics of near IR polymethine dyes. Chem. Phys..

[51] Li D.D., Tian X.H., Wang A.D., Guan L.J., Zheng J., Li F., Li S.L., Zhou H.P., Wua J.Y., Tian Y.P. (2016). Nucleic acid-selective light-up fluorescent biosensors for ratiometric two-photon imaging of the viscosity of live cells and tissues. Chem. Sci..

[52] Chen Y., Bai Y., Han Z., He W., Guo Z. (2015). Photoluminescence imaging of Zn^2+^ in living systems. Chem. Soc. Rev..

[53] Hao F.Y., Zhang X.J., Tian Y.P., Zhou H.P., Li L., Wu J.Y., Zhang S.Y., Yang J.X., Jin B.K., Tao X.T. (2009). Design, crystal structures and enhanced frequency-upconverted lasing efficiencies of a new series of dyes from hybrid of inorganic polymers and organic chromophores. J. Mater. Chem..

[54] Zhou S.S., Zhang Q., Tian X.H., Hu G.J., Hao F.Y., Wu J.Y., Tian Y.P. (2011). Synthesis, crystal structure, optical properties, DNA-binding and cell imaging of an organic chromophore. Dyes Pigments.

[55] Liu Z.D., Hao F.Y., Xu H.J., Wang H., Wu J.Y., Tian Y.P. (2015). A-π-D-π-A pyridinium salts: Synthesis, crystal structures, two-photon absorption properties and application to biological imaging. CrystEngComm.

[56] Li D.D., Zhang Q., Sun X.S., Shao N.Q., Li R., Li S.L., Zhou H.P., Wu J.Y., Tian Y.P. (2014). Hydrosoluble two-photon absorbing materials: A series of sulfonated organic inner salts in biological imaging application. Dyes Pigments.

[57] Li D.D., Yu D.H., Zhang Q., Li S.L., Zhou H.P., Wu J.Y., Tian Y.P. (2013). Synthesis, crystal structure and third-order nonlinear optical properties in the near-IR range of a novel stilbazolium dye substituted with flexible polyether chains. Dyes Pigments.

[58] Zhang Q., Luo L., Xu H., Hu Z.J., Brommesson C., Wu J.Y., Sun Z.Q., Tian Y.P., Uvdal K. (2016). Design, synthesis, linear and nonlinear photophysical properties of novel pyrimidine-based imidazole derivatives. New J. Chem..

[59] Huang Z.L., Lei H., Li N., Qiu Z.R., Wang H.Z., Guo J.D., Luo Y., Zhong Z.P., Liu X.F., Zhou Z.H. (2003). Novel heterocycle-based organic molecules with two-photon induced blue fluorescent emission. J. Mater. Chem..

[60] Wei P., Bi X.D., Wu Z., Xu Z. (2005). Synthesis of Triphenylamine-Cored Dendritic Two-Photon Absorbing Chromophores. Org. Lett..

[61] Li L., Tian Y.P., Yang J.X., Sun P.P., Wu J.Y., Zhou H.P., Zhang S.Y., Jin B.K., Xing X.J., Wang C.K. (2009). Facile Synthesis and Systematic Investigations of a Series of Novel Bent-Shaped Two-Photon Absorption Chromophores Based on Pyrimidine. Chem. Asian J..

[62] Wang H., Zhang Q., Zhang J., Li L., Zhang Q., Li S.L., Zhang S.Y., Wu J.Y., Tian Y.P. (2014). Synthesis, two-photon absorption properties and bio-imaging applications of mono-, di- and hexa-branched pyrimidine derivatives. Dyes Pigments.

[63] Belfield K.D., Bondar M.V., Morales A.R., Frazer A., Mikhailov I.A., Przhonska O.V. (2013). Photophysical properties and ultrafast excited-state dynamics of a new twophoton absorbing thiopyranyl probe. J. Phys. Chem. C.

[64] Xie Y.P., Zhang X.F., Zhang Y.D., Zhou F., Qi J., Qu J.L. (2012). Fusing three perylenebisimide branches and a truxene core into a star-shaped chromophore with strong two-photon excited fluorescence and high photostability. Chem. Commun..

[65] Zhang Q., Tian X.H., Wang H., Hu Z.J., Wu J.Y., Zhou H.P., Zhang S.Y., Yang J.X., Sun Z.Q., Tian Y.P. (2016). NIR-region two-photon fluorescent probes for Fe^3+^/Cu^2+^ ions based on pyrimidine derivatives with different flexible chain. Sens. Actuators B.

[66] Tang C., Zhang Q., Li D.D., Zhang J., Shi P.F., Li S.L., Wu J.Y., Tian Y.P. (2013). Synthesis, crystal structures, two-photon absorption and biological imaging application of two novel bent-shaped pyrimidine derivatives. Dyes Pigments.

[67] Chung S.J., Kim K.S., Lin T.C., He G.S., Swiatkiewicz J., Prasad P.N. (1999). Cooperative Enhancement of Two-Photon Absorption in Multi-branched Structures. J. Phys. Chem. B.

[68] Paek S.H., Cho N., Cho S., Lee J.K., Ko J.J. (2012). Planar Star-Shaped Organic Semiconductor with Fused Triphenylamine Core for Solution-Processed Small-Molecule Organic Solar Cells and Field-Effect Transistors. Org. Lett..

[69] Liang M., Xu W., Cai F., Chen P., Peng B., Chen J., Li Z. (2007). New Triphenylamine-Based Organic Dyes for Efficient Dye-Sensitized Solar Cells. J. Phys. Chem. C.

[70] Zhu M.R., Zou J.H., He X., Yang C.L., Wu H.B., Zhong C., Qin J.G., Cao Y. (2012). Triphenylamine Dendronized Iridium(III) Complexes: Robust Synthesis, Highly Efficient Nondoped Orange Electrophosphorescence and the Structure–Property Relationship. Chem. Mater..

[71] Andrade C.D., Yanez C.O., Qaddoura M.A., Wang X., Arnett C.L., Coombs S.A., Yu J., Bassiouni R., Bondar M.V., Belfield K.D. (2011). Two-Photon Fluorescence Lysosomal Bio-imaging with a Micelle-Encapsulated Fluorescent Probe. J. Fluoresc..

[72] Xie D., Jing J., Cai Y.B., Tang J., Chen J.J., Zhang J.L. (2014). Construction of an orthogonal ZnSalen/Salophen library as a colour palette for one- and two-photon live cell imaging. Chem. Sci..

[73] Liu B., Zhang Q., Ding H.J., Hu G.J., Du Y.J., Wang C.K., Wu J.Y., Li S.L., Zhou H.P., Yang J.X. (2012). Synthesis, crystal structures and two-photon absorption properties of a series of terpyridine-based chromophores. Dyes Pigments.

[74] Liu J., Zhu Y.Z., Tian X.H., Li F., Xu W.T., Zhang Y.J., Wang C.K., Zhang J., Zhou H.P., Wu J.Y. (2016). Synthesis, crystal structures of a series of novel 2,2′:6′,2″-terpyridine derivatives: The influences of substituents on their photophysical properties and intracellular acid organelle targeting. Dyes Pigments.

[75] Li S.L., Shen W.B., Hu G.J., Zhang Q., Liu B., Liu J., Ding H.J., Wu J.Y., Zhou H.P., Yang J.X. (2013). Crystal structure, nonlinear optical and photophysical properties of a novel chromophore constructed with terpyridine, triphenylamine and ethyl cyanocaetate functional moieties. Mater. Chem. Physi..

[76] Liu Y.Q., Kong M., Zhang Q., Zhang Z.W., Zhou H.P., Zhang S.Y., Li S.L., Wu J.Y., Tian Y.P. (2014). A series of triphenylamine-based two-photon absorbing materials with AIE property for biological Imaging. J. Mater. Chem. B.

[77] Gan X.P., Wang Y., Ge X.P., Li W., Zhang X.Z., Zhu W.J., Zhou H.P., Wu J.Y., Tian Y.P. (2015). Triphenylamine isophorone derivatives with two photon absorption:Photo-physical property, DFT study and bio-imaging. Dyes Pigments.

[78] Macak P., Luo Y., Norman P., Agren H. (2000). Electronic and vibronic contributions to two-photon absorption of molecules with multi-branched structures. J. Chem. Phys..

[79] Zhang Q., Tian X.H., Hu Z.J., Brommesson C., Wu J.Y., Zhou H.P., Yang J.X., Sun Z.Q., Tian Y.P., Uvdal K. (2016). Nonlinear optical response and two-photon biological applications of a new family of imidazole-pyrimidine derivatives. Dyes Pigments.

[80] Yu Z.P., Zheng Z., Yang M.D., Wang L.K., Tian Y.P., Wu J.Y., Zhou H.P., Xu H.M., Wu Z.Q. (2013). Photon-induced intramolecular charge transfer with the influence of D/A group and mode: Optical physical properties and bio-imaging. J. Mater. Chem. C.

[81] Tian Y.P., Li L., Zhang J.Z., Yang J.X., Zhou H.P., Wu J.Y., Sun P.P., Tao L.M., Gao Y.H., Wang C.K. (2007). Investigations and facile synthesis of a series of novel multi-functional two-photon absorption materials. J. Mater. Chem..

[82] Murphy S., Huang L., Kamat P.V. (2011). Charge-Transfer Complexation and Excited-State Interactions in Porphyrin-Silver Nanoparticle Hybrid Structures. J. Phys. Chem. C.

[83] Kometani N., Tsubonishi M., Fujita T., Asami K., Yonezawa Y. (2001). Preparation and Optical Absorption Spectra of Dye-Coated Au, Ag, and Au/Ag Colloidal Nanoparticles in Aqueous Solutions and in Alternate Assemblies. Langmuir.

[84] Nabika H., Takase M., Nagasawa F., Murakoshi K. (2010). Toward Plasmon-Induced Photoexcitation of Molecules. J. Phys. Chem. Lett..

[85] Lee J., Hernandez P., Lee J.W., Govorov A.O., Kotov N.A. (2007). Exciton–plasmon interactions in molecular spring assemblies of nanowires and wavelength-based protein detection. Nat. Mater..

[86] Gao Y.H., Wu J.Y., Zhao Q., Zheng L.X., Zhou H.P., Zhang S.Y., Yang J.X., Tian Y.P. (2009). Solvent-resolved fluorescent Ag nanocrystals capped with a novel terpyridine-based dye. New J. Chem..

[87] Kim K.S., Kim J.H., Kim H., Laquai F., Lee J.K., Yoo S., Sohn B.H. (2012). Switching off FRET in the Hybrid Assemblies of Diblock Copolymer Micelles, Quantum Dots, and Dyes by Plasmonic Nanoparticles. ACS Nano.

[88] Cohanoschi I., Hernandez F.E. (2005). Surface Plasmon Enhancement of Two- and Three-Photon Absorption of Hoechst 33 258 Dye in Activated Gold Colloid Solution. J. Phys. Chem. B.

[89] Stellacci F., Bauer C.A., Meyer-Friedrichsen T., Wenseleers W., Marder S.R., Perry J.W. (2003). Ultrabright Supramolecular Beacons Based on the Self-Assembly of Two-Photon Chromophores on Metal Nanoparticles. J. Am. Chem. Soc..

[90] Wenseleers W., Stellacci F., Meyer-Friedrichsen T., Mangel T., Bauer C.A., Pond S.J.K., Marder S.R., Perry J.W. (2002). Five Orders-of-Magnitude Enhancement of Two-Photon Absorption for Dyes on Silver Nanoparticle Fractal Clusters. J. Phys. Chem. B.

[91] Lal M., Levy L., Kim K.S., He G.S., Wang X., Min Y.H., Pakatchi S., Prasad P.N. (2000). Silica Nanobubbles Containing an Organic Dye in a Multilayered Organic/Inorganic Heterostructure with Enhanced Luminescence. Chem. Mater..

[92] Kim S., Pudavar H.E., Prasad P.N. (2006). Dye-concentrated organically modified silica nanoparticles as a ratiometric fluorescent pH probe by one- and two-photon excitation. Chem. Commun..

[93] Kim S., Zheng Q.D., He G.S., Bharali D.J., Pudavar H.E., Baev A., Prasad P.N. (2006). Aggregation-Enhanced Fluorescence and Two-Photon Absorption in Nanoaggregates of a 9,10-Bis[4′-(4″-aminostyryl)styryl]anthracene Derivative. Adv. Funct. Mater..

[94] Erogbogbo F., Yong K.T., Roy I., Xu G.X., Prasad P.N., Swihart M.T. (2008). Biocompatible Luminescent Silicon Quantum Dots for Imaging of Cancer Cells. ACS Nano.

[95] Zhang X.J., Tian Y.P., Jin F., Wu J.Y., Xie Y., Tao X.T., Jiang M.H. (2005). Self-Assembly of An Organic Chromophore with Cd−S Nanoclusters: Supramolecular Structures and Enhanced Emissions. Cryst. Growth Des..

[96] Gao Y.H., Wu J.Y., Li Y.M., Sun P.P., Zhou H.P., Yang J.X., Zhang S.Y., Jin B.K., Tian Y.P. (2009). A Sulfur-Terminal Zn(II) Complex and Its Two-Photon Microscopy Biological Imaging Application. J. Am. Chem. Soc..

[97] Constable E.C. (2007). 2,2′:6′,2″-Terpyridines: From chemical obscurity to common supramolecular motifs. Chem. Soc. Rev..

[98] Shirota Y., Kageyama H. (2007). Charge Carrier Transporting Molecular Materials and Their Applications in Devices. Chem. Rev..

[99] Sun W., Wu N.N., Wang W.D., Kong L., Wu J.Y., Li S.L., Tian Y.P. (2016). NIR laser induced TPA enhancement of Zn(II)-terpyridine capped gold nanoparticles for targeting mitochondria. Dyes Pigments.

[100] Li L., Tian Y.P., Yang J.X., Sun P.P., Kong L., Wu J.Y., Zhou H.P., Zhang S.Y., Jin B.K., Tao X.T. (2010). Two-photon absorption enhancement induced by aggregation with accurate photophysical data: Spontaneous accumulation of dye in silica nanoparticles. Chem. Commun..

[101] Picot A., D’Aléo A., Baldeck P.L., Grichine A., Duperray A., Andraud C., Maury O. (2008). Long-Lived Two-Photon Excited Luminescence of Water-Soluble Europium Complex: Applications in Biological Imaging Using Two-Photon Scanning Microscopy. J. Am. Chem. Soc..

[102] Sun Y.G. (2010). Conversion of Ag Nanowires to AgCl Nanowires Decorated with Au Nanoparticles and Their Photocatalytic Activity. J. Phys. Chem..

[103] Chen D.L., Yoo S.H., Huang Q.S., Ali G., Cho S.O. (2012). Sonochemical Synthesis of Ag/AgCl Nanocubes and Their Efficient Visible-Light-Driven Photocatalytic Performance. Chem. Eur. J..

[104] Tagar Z.A., Memon S.N., Agheem M.H., Junejo Y., Hassan S.S., Kalwar N.H., Khattak M.I. (2011). Selective, simple and economical lead sensor based on ibuprofen derived silver nanoparticles. Sens. Actuators B.

[105] Yan J., Han X.J., He J.J., Kang L.L., Zhang B., Du Y.C., Zhao H.T., Dong C.K., Wang H.L., Xu P. (2012). Highly Sensitive Surface-Enhanced Raman Spectroscopy (SERS) Platforms Based on Silver Nanostructures Fabricated on Polyaniline Membrane Surfaces. ACS Appl. Mater. Interfaces.

[106] Papadopoulou E., Bell S.E.J. (2012). Label-Free Detection of Nanomolar Unmodified Single- and Double-Stranded DNA by Using Surface-Enhanced Raman Spectroscopy on Ag and Au Colloids. Chem. Eur. J..

[107] Huang X.H., Neretina S., El-Sayed M.A. (2009). Gold Nanorods: From Synthesis and Properties to Biological and Biomedical Applications. Adv. Mater..

[108] Huang X.H., El-Sayed I.H., Qian W., El-Sayed M.A. (2006). Cancer Cell Imaging and Photothermal Therapy in the Near-Infrared Region by Using Gold Nanorods. J. Am. Chem. Soc..

[109] Zhang J.Z. (2010). Biomedical Applications of Shape-Controlled Plasmonic Nanostructures: A Case Study of Hollow Gold Nanospheres for Photothermal Ablation Therapy of Cancer. Chem. Lett..

[110] Parab H.J., Chen H.M., Lai T., Huang J.H., Chen P.H., Liu R.S., Hsiao M., Chen C., Tsai D., Hwu Y. (2009). Biosensing, Cytotoxicity, and Cellular Uptake Studies of Surface-Modified Gold Nanorods. J. Phys. Chem. C.

[111] Pal S., Tak Y.K., Song J.M. (2007). Does the antibacterial activity of silver nanoparticles depend on the shape of the nanoparticle? A study of the gram-negative bacterium *Escherichia coli*. Appl. Environ. Microbiol..

[112] Singh P., Parent K.L., Buttry D.A. (2012). Electrochemical Solid-State Phase Transformations of Silver Nanoparticles. J. Am. Chem. Soc..

[113] Wang Y.A., Zhang J., Huang L., He D., Ma L., Ouyang J., Jiang F. (2012). Novel Application of Ag Nanoclusters in Fluorescent Imaging of Human Serum Proteins after Native Polyacrylamide Gel Electrophoresis (PAGE). Chem. Eur. J..

[114] Kim H.M., Cho B.R. (2009). Two-Photon Probes for Intracellular Free Metal Ions, Acidic Vesicles, and Lipid Rafts in Live Tissues. Acc. Chem. Res..

[115] Kong B., Zhu A.W., Ding C.Q., Zhao X.M., Li B., Tian Y. (2012). Carbon Dot-Based Inorganic–Organic Nanosystem for Two-Photon Imaging and Biosensing of pH Variation in Living Cells and Tissues. Adv. Mater..

[116] Kong L., Yang J.X., Li S.L., Zhang Q., Xue Z.M., Zhou H.P., Wu J.Y., Jin B.K., Tian Y.P. (2013). A Self-Assembled Nanohybrid Composed of Fluorophore-Phenylamine Nanorods and Ag Nanocrystals: Energy Transfer, Wavelength Shift of Fluorescence and TPEF Applications for Live-Cell Imaging. Chem. Eur. J..

[117] De Sa G.F., Malta O.L., Donega C.D., Simas A.M., Longo R.L., Santa-Cruz P.A., da Silva E.F. (2000). Spectroscopic properties and design of highly luminescent lanthanide coordination complexes. Coord. Chem. Rev..

[118] Binnemans K. (2009). Lanthanide-Based Luminescent Hybrid Materials. Chem. Rev..

[119] Filipescu N., Sager W.F., Serafin F.A. (1964). Substituent Effects on Intramolecular Energy Transfer. II. Fluorescence Spectra of Europium and Terbium *β*-Diketone Chelates. J. Phys. Chem..

[120] Rendell D. (1987). Fluorescence and Phosphorescence Spectroscopy.

[121] Hu Z.J., Tian X.H., Zhao X.H., Wang P., Zhang Q., Sun P.P., Wu J.Y., Yang J.X., Tian Y.P. (2011). Efficient two-photon-sensitized luminescence of a novel europium(III) *β*-diketonate complex and application in biological imaging. Chem. Commun..

[122] Piszczek G., Maliwal B.P., Grycaynski I., Dattelbaum J., Lakowicz J.R., Fluoresc J. (2001). Multiphoton Ligand-Enhanced Excitation of Lanthanides. J. Fluoresc..

[123] Zhou S.S., Xue X., Wang J.F., Dong Y., Jiang B., Wei D., Wan M.L., Jia Y. (2012). Synthesis, optical properties and biological imaging of the rare earth complexes with curcumin and pyridine. J. Mater. Chem..

[124] Wang J.S., Jin F.Z., Ma H.C., Li X.B., Liu M.Y., Kan J.K., Chen G.J., Dong Y.B. (2016). Au@Cu(II)-MOF: Highly Efficient Bifunctional Heterogeneous Catalyst for Successive Oxidation-Condensation Reactions. Inorg. Chem..

[125] Fleetham T.B., Huang L., Klimes K., Brooks J., Li J. (2016). Tetradentate Pt(II) Complexes with 6-Membered Chelate Rings: A New Route for Stable and Efficient Blue Organic Light Emitting Diodes. Chem. Mater..

[126] Garah M.E., Sinn S., Diana A., Santana-Bonilla A., Gutierrez R., De Cola L., Cuniberti G., Ciesielskia A., Samorì P. (2016). Discrete polygonal supramolecular architectures of isocytosine-based Pt(II) complexes at the solution/graphite interface. Chem. Commun..

[127] Romanova J., Prabhath M.R.R., Jarowski P. (2016). Relationship between Metallophilic Interactions and Luminescent Properties in Pt(II) Complexes: TD-DFT Guide for the Molecular Design of Light-Responsive Materials. J. Phys. Chem. C.

[128] Zou H.H., Wang L., Long Z.X., Qin Q.P., Song Z.K., Xie T., Zhang S.H., Liu Y.C., Lin B., Chen Z.F. (2016). Preparation of 4-([2,2′:6′,2″-terpyridin]-4′-yl)-*N*,*N*-diethylaniline NiII and PtII complexes and exploration of their in vitro cytotoxic activities. Eur. J. Med. Chem..

[129] Wawrznek R., Muhieddine K., Ullah M., Koszo P.B., Shaw P.E., Grosjean A., Maasoumi F., Stoltzfus D.M., Clegg J.K., Burn P.L. (2016). Orange-Red-Light-Emitting Field-Effect Transistors Based on Phosphorescent Pt(II) Complexes with Area Emission. Adv. Opt. Mater..

[130] Kantoury M., Moghadam M.E., Tarlani A.A., Divsalar A. (2016). Structure Effect of Some New Anticancer Pt(II) Complexes of Amino Acid Derivatives with Small Branched or Linear Hydrocarbon Chains on Their DNA Interaction. Chem. Biol. Drug Des..

[131] Ho C.L., Yu Z.Q., Wong W.Y. (2016). Multifunctional polymetallaynes: Properties, functions and applications. Chem. Soc. Rev..

[132] Neugebauer U., Pellegrin Y., Devocelle M., Forster R.J., Signac W., Morand N., Keyes T.E. (2008). Ruthenium polypyridyl peptide conjugates: Membrane permeable probes for cellular imaging. Chem. Commun..

[133] Friedman A.E., Chambron J.C., Sauvage J.P., Turro N.J., Barton J.K. (1990). Ru(bpy)2(dppz)2+. J. Am. Chem. Soc..

[134] Jiménez-Hernández M.E., Orellana G., Montero F., Portolés M.T. (2000). A Ruthenium Probe for Cell Viability Measurement Using Flow Cytometry, Confocal Microscopy and Time-resolved Luminescence. Photochem. Photobiol..

[135] Xiao L.F., Wang H., Zhang Q., Zhu Y.Z., Luo J.S., Liang Y.K., Zhang S.Y., Zhou H.P., Tian Y.P., Wu J.Y. (2015). Novel ruthenium(II) polypyridyl complexes containing carbazole with flexible substituents: Crystal structure, nonlinear optical properties and DNA-binding interaction. Dyes Pigments.

[136] Puckett C.A., Barton J.K. (2009). Fluorescein Redirects a Ruthenium–Octaarginine Conjugate to the Nucleus. J. Am. Chem. Soc..

[137] Gill M.R., Garica-Lara J., Foster S.J., Smythe C., Battaglia G., Thomas J.A. (2009). A ruthenium(II) polypyridyl complex for direct imaging of DNA structure in living cells. Nat. Chem..

[138] Wang H., Tian X.H., Guan L.J., Zhang Q., Zhang S.Y., Zhou H.P., Wu J.Y., Tian Y.P. (2016). Targeting mitochondrial DNA with a two-photon active Ru(II) phenanthroline derivative. J. Mater. Chem. B.

[139] Liu J.P., Chen Y., Li G.Y., Zhang P.Y., Jin C.Z., Zeng L.L., Ji L.N., Chao H. (2015). Ruthenium(II) polypyridyl complexes as mitochondria-targeted two-photon photodynamic anticancer agents. Biomaterials.

[140] Zhao Q., Huang C.H., Li F.Y. (2011). Phosphorescent heavy-metal complexes for bio-imaging. Chem. Soc. Rev..

[141] Shi P.F., Coe B.J., Sánchez S., Wang D.Q., Tian Y.P., Nyk M., Samoc M. (2015). Uniting Ruthenium(II) and platimum(II) polypyridine centers in heteropolymetallic complexes giving strong two-photon absorption. Inorg. Chem..

[142] Kaes C., Katz A., Hosseini M.W. (2000). Bipyridine: The Most Widely Used Ligand. A Review of Molecules Comprising at Least Two 2,2′-Bipyridine Units. Chem. Rev..

[143] Hofmeier H., Schubert U.S. (2004). Recent developments in the supramolecular chemistry of terpyridine-metal complexes. Chem. Soc. Rev..

[144] Li D.M., Zhang Q., Wang P., Wu J.Y., Kan Y.H., Tian Y.P., Zhou H.P., Yang J.X., Tao X.T., Jiang M.H. (2011). Studies of the isomerization and photophysical properties of a novel 2,2′:6′,2″-terpyridine-based ligand and its complexes. Dalton Trans..

[145] Zhou F.X., Zheng Z., Zhou H.P., Ke W.Z., Wang J.Q., Yu Z.P., Jin F., Yang J.X., Wu J.Y., Tian Y.P. (2012). A new 2,2′:6′,2″-terpyridine-based ligand and its complexes: Structures, photophysical properties and DFT calculations to evaluate the halogen effect on the TPA. Cryst. Eng. Comm..

[146] Shi P.F., Jiang Q., Zhao X.S., Zhang Q., Tian Y.P. (2015). Study of the one-photon and two-photon properties of two water-soluble terpyridines and their zinc complexes. Dalton Trans..

[147] Albert I.D., Marks T.J., Ratner M.A. (1998). Remarkable NLO Response and Infrared Absorption in Simple Twisted Molecular *π*-Chromophores. J. Am. Chem. Soc..

[148] Righetto S., Rondena S., Locatelli D., Roberto D., Tessore F., Ugo R., Quici S., Roma S., Korystov D., Srdanov V.I. (2006). An investigation on the two-photon absorption activity of various terpyridines and related homoleptic and heteroleptic cationic Zn(II) complexes. J. Mater. Chem..

[149] Ruiz Delgado M.C., Casado J., Hernandez V., López Navarrete J.T., Orduna J., Villacampa B., Alicante R., Raimundo J.M., Blanchard P., Roncali J.J. (2008). Electronic, Optical, and Vibrational Properties of Bridged Dithienylethylene-Based NLO Chromophores. J. Phys. Chem. C.

[150] Colombo A., Locatelli D., Roberto D., Tessore F., Ugo R., Cavazzini M., Quici S., De Angelis F., Fantacci S., Ledoux-Rak I. (2012). New [(D-terpyridine)-Ru-(D or A-terpyridine)][4-EtPhCO2]2 complexes (D = electron donor group; A = electron acceptor group) as active second-order non linear optical chromophores. Dalton Trans..

[151] Schwich T., Cifuentes M.P., Gugger P.A., Samoc M., Humphrey M.G. (2011). Electronic, Molecular Weight, Molecular Volume, and Financial Cost-Scaling and Comparison of Two-Photon Absorption Efficiency in Disparate Molecules (Organometallic Complexes for Nonlinear Optics. 48.)—A Response to “Comment on ‘Organometallic Complexes for Nonlinear Optics. 45. Dispersion of the Third-Order Nonlinear Optical Properties of Triphenylamine-Cored Alkynylruthenium Dendrimers’. Increasing the Nonlinear Response by Two Orders of Magnitude”. Adv. Mater..

[152] Roberts R.L., Schwich T., Corkery T.C., Cifuentes M.P., Green K.A., Farmer J.D., Low P.J., Marder T.B., Samoc M., Humphrey M.G. (2009). Organometallic Complexes for Nonlinear Optics. 45. Dispersion of the Third-Order Nonlinear Optical Properties of Triphenylamine-Cored Alkynylruthenium Dendrimers. Adv. Mater..

[153] Tan J.Y., Li R., Li D.D., Zhang Q., Li S.L., Zhou H.P., Yang J.X., Wu J.Y., Tian Y.P. (2015). Thiophene-based terpyridine and its zinc halide complexes: Third-order nonlinear optical properties in the near-infrared region. Dalton Trans..

[154] Schlutter F., Wild A., Winter A., Hager M.D., Baumgaertel A., Friebe C., Schubert U.S. (2010). Synthesis and Characterization of New Self-Assembled Metallo-Polymers Containing Electron-Withdrawing and Electron-Donating Bis(terpyridine) Zinc(II) Moieties. Macromolecules.

[155] Schmittel M., Kalsani V., Kishore R.S., Cölfen H., Bats J.W. (2005). Dynamic and Fluorescent Nanoscale Phenanthroline/Terpyridine Zinc(II) Ladders. Self-Recognition in Unlike Ligand/Like Metal Coordination Scenarios. J. Am. Chem. Soc..

[156] He G.S., Tan L.S., Zheng Q., Prasad P.N. (2008). Multiphoton Absorbing Materials: Molecular Designs, Characterizations, and Applications. Chem. Rev..

[157] Zhang Q., Tian X.H., Hu Z.J., Brommesson C., Wu J.Y., Zhou H.P., Li S.L., Yang J.X., Sun Z.Q., Tian Y.P. (2015). A series of Zn(II) terpyridine complexes with enhanced two-photon-excited fluorescence in vitro and in vivo Bio-imaging. J. Mater. Chem. B.

[158] Rendón N., Bourdolle A., Baldeck P.L., Bozec H.L., Andraud C., Brasselet S., Copéret C., Maury O. (2011). Bright Luminescent Silica Nanoparticles for Two-Photon Microscopy Imaging via Controlled Formation of 4,4′-Diethylaminostyryl-2,2′-bipyridine Zn(II) Surface Complexes. Chem. Mater..

[159] Barzoukas M., Runser C., Fort A., Blanchard-Desce M. (1996). A two-state description of (hyper) polarizabilities of push-pull molecules based on a two-form model. Chem. Phys. Lett..

[160] Tian X.H., Zhang Q., Zhang M.Z., Uvdal K., Wang Q., Chen J.Y., Du W., Huang B., Wu J.Y., Tian Y.P. (2017). Probe for simultaneous membrane and nucleus labeling in living cells and in vivo bio-imaging using a two-photon absorption water-soluble Zn(II) terpyridine complex with a reduced *π*-conjugation system. Chem. Sci..

[161] Li D.M., Tian X.H., Hu G.J., Zhang Q., Wang P., Sun P.P., Zhou H.P., Meng X.M., Yang J.X., Wu J.Y. (2011). Synthesis, Crystal Structures, Photophysical Properties, and Bio-imaging of Living Cells of Bis-*β*-Diketonate Phenothiazine Ligands and Its Cyclic Dinuclear Complexes. Inorg. Chem..

[162] Zhang C., Cao Y., Zhang J.F., Humphrey M.G. (2008). Modulation of Third-Order Nonlinear Optical Properties by Backbone Modification of Polymeric Pillared-Layer Heterometallic Clusters. Adv. Mater..

[163] Ford P.C., Cariati E., Bourassa J. (1999). Photoluminescence Properties of Multinuclear Copper(I) Compounds. Chem. Rev..

[164] Wang X.C., Tian X.H., Zhang Q., Sun P.P., Wu J.Y., Zhou H.P., Jin B.K., Yang J.X., Zhang S.Y., Wang C.K. (2012). Assembly, Two-Photon Absorption and Bio-imaging of Living Cells of A Cuprous Cluster. Chem. Mater..

[165] Katan C., Charlot M., Mongin O., Droumaguet C., Jouikov V., Terenziani F., Badaeva E., Tretiak S., Blanchard-Desce M.J. (2010). Simultaneous Control of Emission Localization and Two-Photon Absorption Efficiency in Dissymmetrical Chromophores. Phys. Chem. B.

[166] Xu C., Zipfel W., Shear J.B., Williams R.M., Webb W.W. (1996). Multiphoton fluorescence excitation: New spectral windows for biological nonlinear microscopy. Proc. Natl. Acad. Sci. USA.

[167] Picot A., Malvolti F., Le Guennic B., Baldeck P.L., Williams J.A.G., Andraud C., Maury O. (2007). Two-Photon Antenna Effect Induced in Octupolar Europium Complexes. Inorg. Chem..

[168] Hai Y., Chen J.J., Zhao P., Lv H.B., Yu Y., Xu P.Y., Zhang J.L. (2011). Luminescent zinc salen complexes as single and two-photon fluorescence subcellular imaging probes. Chem. Commun..

[169] Leydet Y., Bassani D.M., Jonusauskas G., McClenaghan N.D. (2007). Equilibration between Three Different Excited States in a Bichromophoric Copper(I) Polypyridine Complex. J. Am. Chem. Soc..

[170] Bertrand B., Casini A. (2014). A golden future in medicinal inorganic chemistry: The promise of anticancer gold organometallic compounds. Dalton Trans..

[171] Zhang W.Q., Yu J.C., Cui Y.J., Rao X.T., Yang Y., Qian G.D. (2013). Assembly and tunable luminescence of lanthanide-organic frameworks constructed from 4-(3,5-dicarboxyphenyl)pyridine-2,6-dicarboxylate ligand. J. Alloys Compd..

[172] Wang W.H., Muckerman J.T., Fujita E., Himeda Y. (2013). Hydroxy-substituted pyridine-like *N*-heterocycles: Versatile ligands in organometallic catalysis. New J. Chem..

[173] Denard C.A., Huang H., Bartlett M.J., Lu L., Tan Y.C., Zhao H.M., Hartwig J.F. (2014). Cooperative Tandem Catalysis by an Organometallic Complex and a Metalloenzyme. Angew. Chem..

[174] Shaheen F., Ali S., Meetsma A. (2012). Structural properties and antibacterial potency of new supramolecular organotin(IV) dithiocarboxylates. Polyhedron.

[175] Wobser S.D., Stephenson C.J., Delferro M., Marks T.J. (2012). Carbostannolysis Mediated by Bis(pentamethylcyclopentadienyl)lanthanide Catalysts. Utility in Accessing Organotin Synthons. Organometallics.

[176] Tariq M., Ali S., Shah N.A., Muhammad N., Tahir M.N., Khalid N., Khan M.R. (2013). Catalytic, biological and DNA binding studies of organotin(IV) carboxylates of 3-(2-fluorophenyl)-2-methylacrylic acid: Synthesis, spectroscopic characterization and X-ray structure analysis. Polyhedron.

[177] Hadjikakou S.K., Hadjiliadis N. (2009). Antiproliferative and anti-tumor activity of organotin compounds. Coord. Chem. Rev..

[178] Ma C.L., Zhang S.L., Zhang R.F. (2012). Chiral self-assembly of triorganotin complexes: Syntheses, characterization, crystal structures and antitumor activity of organotin(IV) complexes containing (R)-(+)-methylsuccinic acid, (S)-(+)-methylglutaric acid and l-(−)-malic acid ligands. Polyhedron.

[179] Chandrasekhar V., Mohapatra C., Banerjee R., Mallick A. (2013). Synthesis, Structure, and H_2_/CO_2_ Adsorption in a Three-Dimensional 4-Connected Triorganotin Coordination Polymer with a sqc Topology. Inorg. Chem..

[180] Xiao X., Shao K.Z., Yan L.S., Mei Z.M., Zhu D.S., Xu L. (2013). A novel macrocyclic organotin carboxylate containing a nona-nuclear long ladder. Dalton Trans..

[181] Chandrasekhar V., Mahapatra C. (2013). 2D-Coordination Polymer Containing Interconnected 82-Membered Organotin Macrocycles. Cryst. Growth Des..

[182] Li D.M., Hu R.T., Zhou W., Sun P.P., Kan Y.H., Tian Y.P., Zhou H.P., Wu J.Y., Tao X.T., Jiang M.H. (2009). Synthesis, Structures, and Photophysical Properties of Two Organostannoxanes from a Novel Acrylic Acid Derived from Phenothiazine. Eur. J. Inorg. Chem..

[183] Zhao X.S., Liu J., Wang H., Zou Y., Li S.L., Zhang S.Y., Zhou H.P., Wu J.Y., Tian Y.P. (2015). Synthesis, crystal structures and two-photon absorption properties of triphenylamine cyanoacetic acid derivative and its organooxotin complexes. Dalton Trans..

